# New insights into the regulation of *Cystathionine beta synthase (CBS)*, an enzyme involved in intellectual deficiency in Down syndrome

**DOI:** 10.3389/fnins.2022.1110163

**Published:** 2023-01-09

**Authors:** Pierre Conan, Alice Léon, Noéline Caroff, Claire Rollet, Loubna Chaïr, Jennifer Martin, Frédéric Bihel, Olivier Mignen, Cécile Voisset, Gaëlle Friocourt

**Affiliations:** ^1^INSERM, Université de Brest, EFS, UMR 1078, GGB, Brest, France; ^2^Laboratoire d’Innovation Thérapeutique, UMR 7200, IMS MEDALIS, Faculty of Pharmacy, CNRS, Université de Strasbourg, Illkirch, France; ^3^U1227, Lymphocytes B, Autoimmunité et Immunothérapies, INSERM, Université de Brest, Brest, France

**Keywords:** *CBS*, *DYRK1A*, GSK3β, Akt, NF-κB, pharmacological inhibitor

## Abstract

Down syndrome (DS), the most frequent chromosomic aberration, results from the presence of an extra copy of chromosome 21. The identification of genes which overexpression contributes to intellectual disability (ID) in DS is important to understand the pathophysiological mechanisms involved and develop new pharmacological therapies. In particular, gene dosage of *Dual specificity tyrosine phosphorylation Regulated Kinase 1A* (*DYRK1A*) and of *Cystathionine beta synthase* (*CBS*) are crucial for cognitive function. As these two enzymes have lately been the main targets for therapeutic research on ID, we sought to decipher the genetic relationship between them. We also used a combination of genetic and drug screenings using a cellular model overexpressing *CYS4*, the homolog of *CBS* in *Saccharomyces cerevisiae*, to get further insights into the molecular mechanisms involved in the regulation of CBS activity. We showed that overexpression of *YAK1*, the homolog of *DYRK1A* in yeast, increased *CYS4* activity whereas *GSK3*β was identified as a genetic suppressor of *CBS*. In addition, analysis of the signaling pathways targeted by the drugs identified through the yeast-based pharmacological screening, and confirmed using human HepG2 cells, emphasized the importance of Akt/GSK3β and NF-κB pathways into the regulation of CBS activity and expression. Taken together, these data provide further understanding into the regulation of CBS and in particular into the genetic relationship between *DYRK1A* and *CBS* through the Akt/GSK3β and NF-κB pathways, which should help develop more effective therapies to reduce cognitive deficits in people with DS.

## Introduction

Down syndrome (DS) is the most frequent chromosomic aberration, with a prevalence of one in 650–1,000 live births worldwide. This genetic condition results from the presence of an extra copy of chromosome 21, as first described by Lejeune et al. (1959). The triplication of this chromosome and of its ∼225 genes leads to a complex phenotype that includes particular craniofacial features, hypotonia, cardiac, and digestive defects, high incidence of leukemia, early onset of Alzheimer’s disease and intellectual disability (ID). Although the detailed consequences of the overexpression of all these individual genes is difficult to assess, a few of them have been suggested to be of crucial importance in the development of certain phenotypic aspects (Antonarakis, 2017). Concerning ID, a few genes are considered as highly relevant candidates, among which the Amyloid Precursor Protein (*APP*) (Salehi et al., 2007), the Glutamate Receptor, Ionotropic, Kainate 1 (*GRIK1*) (Valbuena et al., 2019), the Regulator of CAlciNeurin 1 (*RCAN1*) (Dudilot et al., 2020) and the *Dual-specificity tyrosine phosphorylation-Regulated Kinase 1A* (*DYRK1A*) (Altafaj et al., 2013; García-Cerro et al., 2014). So far, DYRK1A has been the main target for therapeutic research, leading to the identification of compounds that inhibit its protein kinase activity and are able to improve cognition in mouse models for DS (Guedj et al., 2009; De la Torre et al., 2014, 2016; Kim H. et al., 2016; Nakano-Kobayashi et al., 2017; Neumann et al., 2018; Nguyen et al., 2018). However, their efficiency in DS patients is limited, showing the need to combine multiple therapies to improve cognitive deficits and more generally the quality of life of DS patients.

More recently, studies of transgenic mouse models have revealed that the triplication of *CBS* gene also contributes to cognitive phenotypes and that *CBS* and *DYRK1A* show epistatic interactions (Maréchal et al., 2019). *CBS* encodes a pyridoxal 5′-phosphate-dependent enzyme that catalyzes the first reaction in the transsulfuration pathway. This pathway leads to the synthesis of cysteine and glutathione (GSH) at the expense of homocysteine and methionine (Jhee and Kruger, 2005). In the brain, CBS is also the major enzyme catalyzing the production of hydrogen sulfide (H_2_S) from L-cysteine (Kimura, 2011) or from the condensation of homocysteine with cysteine (Chen et al., 2004). H_2_S is now considered as a major gasotransmitter in the brain, which plays a role in synaptic transmission (Kamat et al., 2015) and its increased production resulting from CBS triplication has been suggested to contribute to the cognitive phenotype of DS patients (Kamoun, 2001; Kamoun et al., 2003; Szabo, 2020). For this reason, the identification of pharmacological inhibitors of CBS has been an important field of research in the last 10 years. Unfortunately, most of the screening methods used were *in vitro* and have only led to the identification of compounds with relatively low potency and limited selectivity (Asimakopoulou et al., 2013; Thorson et al., 2013, 2015; Zhou et al., 2013; Druzhyna et al., 2016), suggesting that CBS may be difficult to target pharmacologically. We recently developed a new screening method based on the budding yeast *Saccharomyces cerevisiae* which allows the identification of drugs or genes that interfere with the phenotypical consequences of *CYS4* (*CBS* homolog in yeast) overexpression. Using this method, we recently identified four molecules (disulfiram, chloroxine, clioquinol, and nitroxoline), all involved in metallic ion binding (Maréchal et al., 2019; Conan et al., 2022), which effect on CBS activity has been validated in different cellular models (Zuhra et al., 2020; Conan et al., 2022).

A genetic interaction between *CBS* and *DYRK1A* has been previously suggested in mouse (Tlili et al., 2013; Latour et al., 2015; Baloula et al., 2018; Maréchal et al., 2019) but the nature of this interaction was still undetermined. It was described as positive in certain studies and negative in others depending on the context or the organ (liver vs. brain). Here, we took advantage of our yeast-based model to explore the relationship between *CBS* and *DYRK1A* genes, which allowed us to confirm the positive regulation of Cbs activity by Dyrk1A. Next, we further investigated the molecular mechanisms involved in the regulation of CBS activity using a combined genetic and drug screening approach. Our results highlighted the importance of the Akt/GSK3β and the NF-κB pathways in the regulation of CBS activity and expression.

## Materials and methods

### Genetic and drug screening in *S. cerevisiae*

**FIGURE 1:**
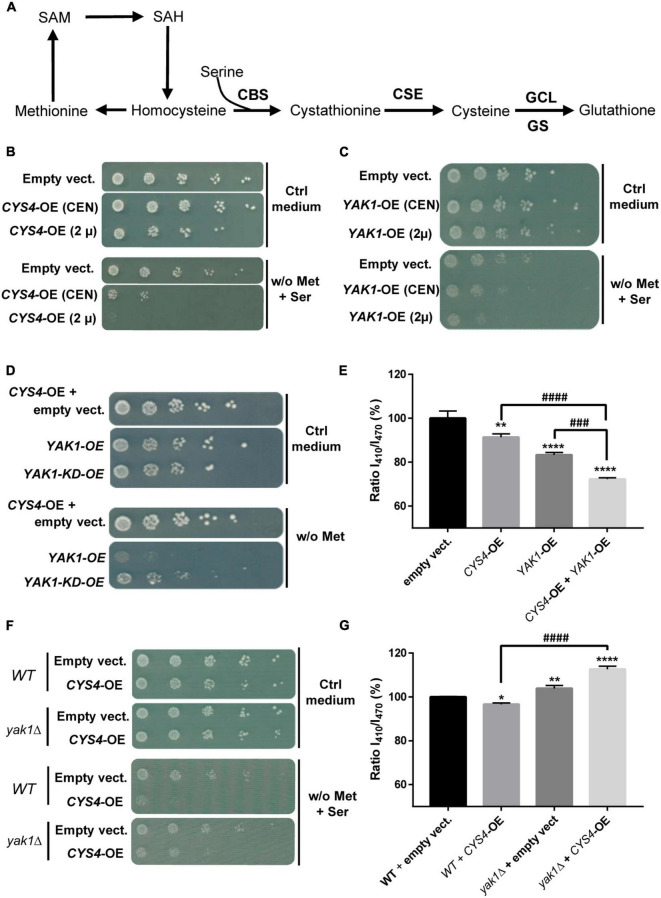
Interaction between *CYS4* and *YAK1*. **(A)** Simplified representation of the transsulfuration pathway. Yeast *CYS4* gene encodes the cystathionine beta synthase protein (CBS) which converts homocysteine and serine into cystathionine. The other enzymes of the transsulfuration pathway are CSE, GCL (γ-glutamylcysteine synthetase), and GS (glutathione synthetase). *CYS4/CBS* overexpression would favor cysteine and glutathione synthesis at the expense of homocysteine and methionine. **(B)** Methionine auxotrophy of *CYS4*-overexpressing (*CYS4*-OE) cells. Methionine auxotrophy, revealed by the absence of growth on medium lacking methionine, was assessed by spotting serial dilutions of wild-type yeast cells transformed with two centromeric (*CEN*) or 2 μ plasmids either empty or containing full length *CYS4*, which expression in yeast is driven by the strong GPD promoter. The presence of a centromere segment (*CEN*) in plasmids enhances their mitotic stability but results in a lower copy number than 2 μ plasmids, thus producing less proteins. **(C)** Methionine auxotrophy of *YAK1*-OE cells. Similarly to *CYS4*-OE, *YAK1*-OE leads to methionine auxotrophy using either a single centromeric plasmid (*CEN*) or a single 2 μ plasmid. **(D,E)** Additive effect of *YAK1*-OE and *CYS4*-OE. **(D)** *YAK1*-OE (obtained with one single 2 μ plasmid) enhances methionine auxotrophy of *CYS4*-OE cells (obtained with two 2 μ plasmids). This effect depends on the kinase activity of *YAK1* as a kinase dead form of *YAK1* (*YAK1*-KD) was not able to strengthen *CYS4*-OE induced phenotype. Note that we used in these experiments a methionine-free medium without serine supplementation to be able to see stronger methionine auxotrophy than the one caused by *CYS4*-OE. **(E)** *YAK1* and *CYS4* overexpression have additive effects on cytosolic acidification. **(F,G)** *YAK1* deletion prevents *CYS4*-OE induced phenotypes. **(F)** *YAK1* deletion mitigates *CYS4*-OE induced methionine auxotrophy. **(G)** Similarly, *CYS4*-OE is not able to induce acidification defects in cells deleted for *YAK1* suggesting that Yak1p is necessary for Cys4p activity. Note that *YAK1* deletion even increases cytosolic pH, suggesting a decrease in GSH synthesis and/or the presence of oxidative stress which may deplete intracellular glutathione. **(E,G)** One-way ANOVA with Tukey’s *post-hoc* test. Comparison with DMSO: *p < 0.05; **p < 0.01; ***p < 0.001; ****p < 0.0001. Comparison between conditions: ^###^*p* < 0.001; ^####^*p* < 0.0001.

*Saccharomyces cerevisiae* strains used in this study are listed in Supplementary Table 1 and were cultured as previously described (Maréchal et al., 2019). Cultures in exponential growth phase, obtained by diluting overnight cultures and incubation for 4–5 h to reach OD_600_∼0.6–1, were used in all experiments. Subcloning of full-length cDNA of *CYS4* in expression vectors of the pRS42X series was performed as previously described (Maréchal et al., 2019) using primers listed in Supplementary Table 2. To obtain a sufficient level of methionine auxotrophy, in all the figures presented (except Figure 1B, in which 2 centromeric plasmids were used), *CYS4* overexpression was obtained through the transfection of two 2 μ vectors of the pRS42X series and the addition in the medium of serine (at a final concentration of 1.5 mM), a limiting substrate for Cys4p activity.

To test the genetic interaction between *YAK1* and *CYS4*, the coding sequence of *YAK1* was amplified from the genomic DNA of a W303 wild-type strain and subcloned into either a pRS416-*GPD* (centromeric plasmid) or a pRS426-*GPD* (2 μ) vector using primers listed in Supplementary Table 2. *YAK1* overexpression was obtained through the transfection of only one vector of the pRS42X series (2 μ plasmids).

For the genetic screening, a yeast genomic DNA library (Lista et al., 2017) constructed by inserting ∼4 kb genomic DNA fragments (obtained by *Sau3A* partial digestion) at the unique *Bam*HI site in the replicative 2 μ multicopy pFL44L vector containing *URA3*-marker, was transformed into a yeast strain overexpressing *CYS4* (with pRS423 and pRS424 plasmids). Transformants were selected on solid minimal medium lacking tryptophan, histidine, uracil, and methionine and supplemented with 1.5 mM of serine. Plasmids originated from the pFL44L-based library were extracted and purified with the Zymoprep kit (Zymo Research), amplified in *Escherichia coli* and then retransformed into the yeast strain overexpressing *CYS4* to confirm their ability to reverse methionine auxotrophy. The extremities of the confirmed clones were sequenced using primers listed in Supplementary Table 2. The coding sequences of genes obtained in the genetic screen were amplified from the pFL44L plasmids extracted from the library and subcloned into pRS426-*TEF* (2 μ) plasmids using primers listed in Supplementary Table 2, which introduced *Sma*I and *Xho*I restriction sites. Yeast deletion of *MCK1* in the W303 background was performed by standard one-step gene replacement with PCR-generated cassettes (Longtine et al., 1998) using primers listed in Supplementary Table 2.

The *YAK1*-KD (kinase dead, p.K398R) and *MCK1*-KD (p.K68R) mutants were created by site-directed mutagenesis (QuickChange Lightning, Agilent technologies, Santa Clara, CA, USA) according to the manufacturer instructions using primers listed in Supplementary Table 2.

Drug screening was performed as previously described (Maréchal et al., 2019; Conan et al., 2022) using compounds obtained from the NIH from different sets: 166 FDA-approved Oncology Drugs (at a concentration of 10 mM in DMSO), 1,584 compounds of the NCI Diversity Set VI (at a concentration of 10 mM in DMSO), 811 compounds of the Mechanistic Set VI (at a concentration of 1 mM in DMSO) and 390 compounds of the Natural Products Set V (at a concentration of 10 mM in DMSO).

Determination of yeast cytosolic pH was performed as previously described (Conan et al., 2022) using a pRS416-*ADH* plasmid containing a pH-sensitive ratiometric GFP variant named pHluorin (kindly obtained from S. Léon, IJM, Paris).

### Cell culture and drug treatment

The human liver cancer cell line HepG2 was obtained from ATCC and was cultured in DMEM glutamax high glucose medium (Invitrogen) supplemented with 10% fetal bovine serum and 100 U/mL penicillin/streptomycin (Invitrogen), in a humidified incubator at 37°C and 5% CO_2_ atmosphere.

All the molecules used in this study were either purchased from Merck or obtained from the NIH libraries and were resuspended in DMSO. For drug treatment, 20,000 HepG2 cells were plated in each well of a Greiner Bio-One black 96-well plate with transparent flat bottom in 100 μL of culture medium. The following day, cells were incubated for 24 or 48 h with selected drugs at indicated concentrations with a final concentration of 1% DMSO (v/v).

### Measurement of H_2_S production in live cells

For HepG2 transfection, 250,000 cells were seeded per well in 6 well plates 24 h before transfection. Cells were then transfected with either the pcDNA3 vector (Invitrogen, Waltham, MA, USA) alone as a negative control or with *GSK3*β or *DYRK1A* cDNA using JetOptimus transfection reagent (Polyplus transfection, Illkirch, France) following the manufacturer’s instructions. The *GSK3*β plasmid was obtained from Addgene (#14754) and corresponds to a constitutively active enzyme (mutant p.S9A). Wild-type and mutant p.S324R and p.S311F *DYRK1A* plasmids were obtained from Courraud et al. (2021). These two mutations, located in the kinase domain of DYRK1A affect its kinase activity (Courraud et al., 2021). Fourty-eight or 72 h after transfection, H_2_S production was assessed as followed.

Following a 24 h-treatment, cells were washed once with 1× PBS and incubated for 2 h in a saline buffer (139 mM NaCl, 0.56 mM MgCl_2_, 10 mM Hepes, 2.7 mM KCl, 1 mM K_2_HPO_4_, 1.8 mM CaCl_2_ pH7.4 supplemented with 10 mM glucose) containing 100 μM of 7-Azido-4-Methylcoumarin (AzMC) fluorescent probe (Sigma Aldrich), which selectively reacts with H_2_S to form a fluorescent compound. Fluorescent AzMC signal acquisition (λ_Ex_ = 365 nm and λ_Em_ = 450 nm) was performed on a Flexstation 3 microplate reader using the SoftMax Pro 5.4.5 software (Molecular Device, San Josa, CA, USA). Values were expressed as a percent of the corresponding controls.

### Cell viability assessment

The cytotoxicity of all tested compounds was examined using the Cell Counting Kit WST-8/CCK8 (Abcam). Briefly, following the measurement of H_2_S levels, cells were washed once with 1× PBS and incubated for 2 h in the WST-8 reagent mixed in the culture medium according to the manufacturer’s instructions. The absorbance signal acquisition (at 450 nm) was performed on the Flexstation 3 microplate reader with SoftMax Pro 5.4.5 software (Molecular Device, San Josa, CA, USA). Values were expressed as a percent of the corresponding controls.

### Western blot

HepG2 cells were treated for 24 or 48 h with selected drugs and were harvested in the following buffer: 150 mM NaCl, 1% Igepal, 50 mM Tris–HCl pH 7.4 with Protease inhibitor cocktail (Roche). Cell lysis was then performed by 6 cycles of vigorous vortexing and freeze-thawing. Protein amount in the supernatants was evaluated by classical Bradford method. Twenty micrograms of each sample were then loaded onto 10% NuPAGE Bis-Tris gels (precast NuPAGE, Invitrogen), and transferred onto 0.45 μm nitrocellulose membranes (Cytiva, Velizy-Villacoublay, France). Membranes were blocked during 1 h at room temperature in 1× PBS containing 0.1% Igepal and 5% milk and then incubated overnight at 4°C with the following primary antibodies: anti-CBS mouse monoclonal antibody (ab12476, Abcam, 1:1,000) and anti-α-tubulin mouse monoclonal antibody (T6793, Sigma-Aldrich, 1:10,000). The following day, membranes were washed with fresh 1× PBS with 0.1% Igepal and incubated for 45 min with gaot anti-mouse (ab6789, Abcam, 1:3,000) conjugated to horseradish peroxidase at a 1:3,000 dilution, and analyzed by enhanced chemiluminescence (ECL, Cytiva) using a Vilbert-Lourmat Photodocumentation Chemistart 5000 imager.

### RT-qPCR

HepG2 cells were treated for 24 h and RNA was isolated using the NucleoSpin RNA mini kit (Macherey-Nagel) and reverse transcribed using RT^2^ First Strand Kit (SA Biosciences) following the manufacturers’ instructions. Real-Time PCR was performed on an *ABI* PRISM^®^ 7300 Sequence Detection System using RT^2^ SYBR Green ROX qPCR Mastermix (Qiagen). Expression levels were normalized across samples using the GAPDH or β-actin housekeeping genes. Primers used are listed in Supplementary Table 3. Data analysis was done by the 2^–ΔΔCt^ method and relative expression was calculated using DMSO condition as the reference sample (expression = 1). Significant differences were assessed by the unpaired two-tailed *t*-test.

### Statistical analysis

Statistical analysis was performed using the GraphPad Prism software (San Diego, CA, USA). Presented results in each figure represent data obtained in at least 3 independent experiments.

## Results

### Yak1p promotes Cys4p activity through its kinase activity

We first investigated the relationship between CBS and DYRK1A using an original yeast-based assay that we recently set-up to identify CBS inhibitors (Maréchal et al., 2019; Conan et al., 2022), and which is based on the overexpression of *CYS4*, the homolog of *CBS* in *S. cerevisiae*. Located at a metabolic hub, CBS/Cys4p converts homocysteine and serine into cystathionine (Figure 1A). As previously shown (Maréchal et al., 2019; Conan et al., 2022), *CYS4*-overexpression (OE) in yeast favors cysteine and glutathione synthesis at the expense of methionine and homocysteine (Figure 1A), leading to a decreased ability of yeast cells to grow without external supply of methionine (Figure 1B). This effect can be enhanced by the addition in the medium of serine, a substrate of the reaction, making this phenotype a convenient read-out that can be easily monitored and used for drug or genetic screening.

Similarly to what was observed for *CYS4-*OE, the overexpression of *YAK1*, the homolog of *DYRK1A* in *S. cerevisiae*, induced by itself methionine auxotrophy in a dose-dependent manner on medium supplemented with serine (Figure 1C). In addition, simultaneous *YAK1*-OE and *CYS4-*OE showed additive effect on methionine auxotrophy (Figure 1D). Yak1p activity appeared to be mediated by its kinase activity as its effect was lost when a kinase dead (KD) form p.K398R of *YAK1* (Moriya et al., 2001) was used (Figure 1D). We previously identified cytosolic acidification as another phenotype specific to *CYS4*-OE (Conan et al., 2022). We show here that, similarly to *CYS4-OE*, *YAK1-OE* induced cytosolic acidification and that combined effect of *CYS4-OE* and *YAK1*-OE on cytosolic pH was additive (Figure 1E). On the opposite, *YAK1* deletion partially rescued methionine auxotrophy due to *CYS4-OE* (Figure 1F). Similarly, *yak1*Δ cells showed increased cytosolic pH, suggestive of an absence of Cys4p activity as shown by Conan et al. (2022). Finally, *CYS4-*OE in a *yak1*Δ strain was unable to induce cytoplasmic acidification defects and even further accentuated cytosolic pH basification (Figure 1G). Taken together, these results suggest that Yak1p promotes Cys4p activity through its kinase activity and that, in the absence of Yak1p, Cys4p activity is reduced.

### Identification of *MCK1*, the yeast homolog of *GSK3*, as a genetic suppressor of *CYS4*-OE phenotypes

**FIGURE 2:**
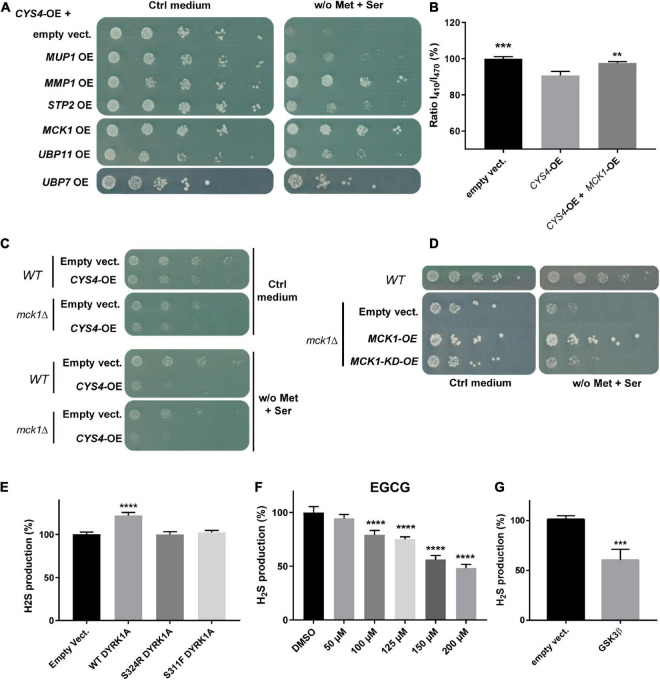
A genetic screening identified *MCK1* as a genetic repressor of *CYS4*-OE induced phenotypes. **(A,B)** *MCK1*-OE rescues the consequences of *CYS4*-OE. **(A)** Genes that were the most effective in rescuing the methionine auxotrophy induced by *CYS4*-OE include genes related to amino acid import (*MUP1*, *MMP2*, *STP2*), a kinase (*MCK1*), and two genes encoding deubiquitinating enzymes (*UBP7* and *UBP11*). **(B)** *MCK1*-OE restores normal cytosolic acidification of *CYS4*-OE cells. Comparison of each condition with *CYS4*-OE, one-way ANOVA with Dunnett’s *post-hoc* test: **p < 0.01; ***p < 0.001. **(C,D)** Deletion of *MCK1* causes slight methionine auxotrophy. **(C)** Deletion of *MCK1* strengthens the effect of *CYS4*-OE on methionine auxotrophy. **(D)** The m*ck1*Δ strain, similarly to *CYS4*-OE, has difficulty to grow on a methionine-free medium complemented with serine, and this methionine auxotrophy is rescued by the expression of wild-type *MCK1* but not of a kinase-dead form of Mck1p (*MCK1-KD*). **(E)** Effect of human *Dual specificity tyrosine phosphorylation Regulated Kinase 1A (DYRK1A)* overexpression on H_2_S production in HepG2 cells. The expression of wild-type Dyrk1A induced increased H_2_S expression 48 h after transfection, effect that was not visible using two mutants p.S324R and p.S311F which affect its kinase activity. **(F)** Inhibition of Dyrk1A activity decreased H_2_S production. A 24 h-treatment with epigallocatechin gallate (EGCG), an inhibitor of Dyrk1A resulted in a dose-dependent decrease in H_2_S production. **(G)** The expression of a constitutively active form of GSK3β (p.S9A) resulted in decreased H_2_S production 72 h after transfection. **(E,F)** Comparison of each condition with empty vector or DMSO, one-way ANOVA with Dunnett’s *post-hoc* test: ****p < 0.0001. **(G)** Student’s *t*-test: ***p < 0.001.

To get better insights into the cellular mechanisms involved in *CYS4*-OE induced phenotypes, we sought to identify genetic suppressors. Amongst the genes having the capacity to save the methionine auxotrophy of *CYS4*-OE cells, six genes had a strong effect: *MUP1* (a methionine permease), *MMP1* (a S-methylmethionine permease), *STP2* (a transcription factor that activates the transcription of amino acid permease genes), *MCK1* (one of the four genes that encode glycogen synthase kinase 3 (GSK3) homologs in yeast), and *UBP7* and *UBP11*, two ubiquitin specific proteases involved in endocytosis and in the sorting of internalized receptors (Tardiff et al., 2013; Weinberg and Drubin, 2014; Figure 2A). The overexpression of *MUP1*, *MMP1*, and *STP2* may act in bringing up traces of methionine or related amino acids present in the medium to rescue the methionine auxotrophy induced by *CYS4*-OE. However, as the overexpression of *MUP1* and *MMP1* appears to also decrease cytosolic acidification of *CYS4*-OE cells (Supplementary Figure 1A), their action may also involve other molecular mechanisms. Similarly, *MCK1* (Figure 2B), *UBP11* and *UBP7* (Supplementary Figure 1B) were also able to counteract the effects of *CYS4*-OE on cytosolic acidification.

Then, due to the important role of *MCK1/GSK3* in cell signaling, we focused our attention on this genetic repressor of *CYS4*-OE induced phenotypes. As shown in Figures 2A, B *MCK1*-OE counteracted both methionine auxotrophy and cytosolic acidification phenotypes induced by *CYS4*-OE. On the opposite, *MCK1* deletion appeared to slightly enhance the methionine auxotrophy of *CYS4*-OE cells (Figure 2C), suggesting that in the absence of Mck1p, Cys4p activity may be promoted. This hypothesis is further strengthened by the fact that a *mck1*Δ strain (without *CYS4* overexpression) shows slight methionine auxotrophy, which is rescued by the expression of wild-type *MCK1* but not by a KD form of *MCK1* (p.K68R, Lim et al., 1993; Figure 2D). Taken together, these results thus show that Cys4p activity is reduced in the presence of Mck1p but increased in the absence of Mck1p, suggesting that Mck1p inhibits directly or not Cys4p activity, and that Mck1p kinase activity is required.

### The genetic interactions between *CYS4* and *YAK1*, and between *CYS4* and *MCK1*, are conserved in mammalian cells

Then, to check whether the regulation of Cys4p by Yak1p is conserved in mammalian cells, we tested the effect of the expression of *DYRK1A* in the human hepatoma HepG2 cell line. We chose this cell line because CBS is known to be highly expressed and active in the liver. In addition, this cell line is commonly used to study CBS-mediated hydrogen sulfide (H_2_S) production (Wang et al., 2018), as cystathionine γ lyase (CSE), the other enzyme involved in H_2_S production, is expressed at very low levels in HepG2 cells. H_2_S production, measured using the 7-AzMC fluorogenic probe as previously described (Conan et al., 2022), can be thus considered in HepG2 as a read-out for CBS activity. As shown in Figures 2E, D YRK1A expression in HepG2 cells induced a significant increase in H_2_S production without affecting cell proliferation and/or viability (Supplementary Figure 1C), whereas two mutant forms of *DYRK1A* (p.S324R and p.S311F) that had lost their kinase activity (Courraud et al., 2021) had no effect on H_2_S production (Figure 2E). Conversely, a 24 h treatment with epigallocatechin gallate (EGCG), an inhibitor of DYRK1A, decreased H_2_S production in a dose-dependent manner (Figure 2F and Supplementary Figure 1D). Similarly, the expression of a constitutively activated form of *GSK3*β (p.S9A mutant) in HepG2 cells induced a significant decrease of H_2_S production (Figure 2G) without affecting cell proliferation and/or viability (Supplementary Figure 1E). Taken together, these results confirm genetic interactions between *CBS* and *DYRK1A* and between *CBS* and *GSK3*β, which are conserved between yeast and human.

### Several small molecules identified in a drug screening appear to converge on metal ion chelation and/or inhibition of NF-κB and Akt/GSK3β pro-survival signaling pathways

To get further insights into the molecular mechanisms involved in the regulation of CBS activity, we screened a set of 2,932 small molecules from the National Cancer Institute, consisting in diverse chemical scaffolds, including natural products and approved oncology drugs. Several compounds were able to restore cell growth of *CYS4*-OE cells on medium without methionine (Supplementary Table 4). Remarkably, the vast majority of the molecules identified in this screen have been described to form complexes with metal ions or to either inhibit NF-κB and/or the Akt/GSK3β pathway (Table 1). Interestingly, six of these compounds have the property to form complexes with Cu(II) (Table 1), supporting our previous findings that copper chelation efficiently decreases CBS activity both in yeast and mammalian cells (Conan et al., 2022). Similarly, zinc pyrithione, a zinc ionophore (Ding and Lind, 2009) that we previously described (Conan et al., 2022), was also found active in the drug screening (Table 1 and Supplementary Table 4).

**TABLE 1:** The molecules identified in the screen share common properties such as metal cation binding or target pro-survival pathways.

Compound	Metal-binding properties	Effect on NF-κ B pathway	Effect on Akt/GSK3β pathway	References
9-Methylstreptimidone	None	Inhibitor	possible inhibitor	Wang et al., 2006; Ishikawa et al., 2009; Brassesco et al., 2012; Koide et al., 2015
4-(2-Thiazolylazo)-resorcinol	Cu(II)			Stanley and Cheney, 1966
1-(2-Thiazolylazo)-2-naphtol	Cu(II)			Pease and Williams, 1959
Zinc pyrithione	Zn(II)	Inhibitor		Kim et al., 1999; Ding and Lind, 2009
N,N- dimethyldaunomycin hydrochloride	Cu(II)	Inhibitor		Malatesta et al., 1987; Boland et al., 1997; Ho et al., 2005; Jabłoñska-Trypuæ et al., 2017
Doxorubicin dihydrochloride				
Daunorubicin hydrochloride				
γ-thujaplicin	Cu(II), Zn(II)	Inhibitor	Inhibitor	MacLean and Gardner, 1956; Miyamoto et al., 1998; Byeon et al., 2008; Huang et al., 2015; Wu et al., 2020
Verrucarin A,10-epoxide		Inhibitor	Inhibitor	Deeb et al., 2016; Liu et al., 2016
8α-Hydroxy-verrucarin A				
Monoacetyl verrucarin A epoxide				
Chrysomycin A		Inhibitor	Inhibitor	Liu et al., 2021, 2022
Naphtoquinones	Cu(II)	Inhibitor		Brandelli et al., 2004; Golan-Goldhirsh and Gopas, 2014

**FIGURE 3:**
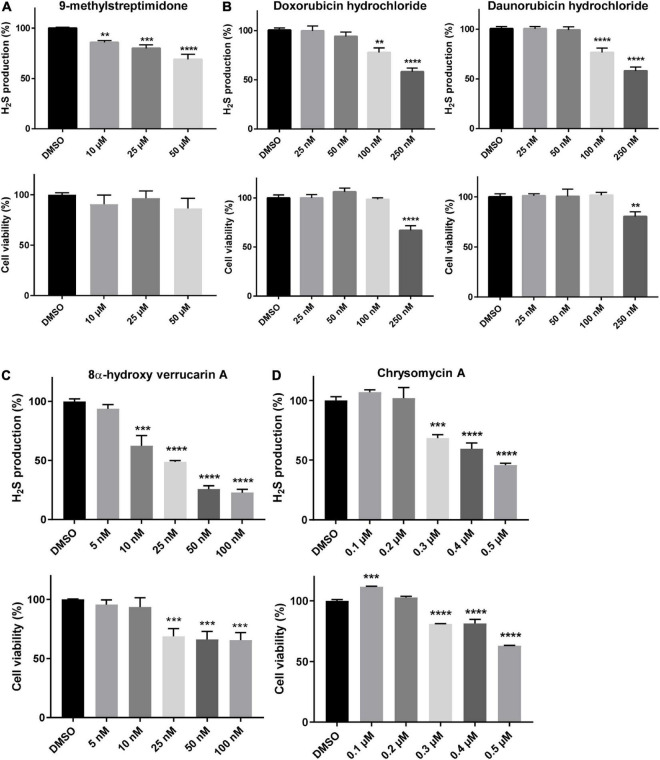
Molecules reducing the phenotypes induced by *CYS4*-OE decrease H_2_S production. The level of H_2_S production and cell viability were assessed using the Azido-4-Methylcoumarin (AzMC) probe and the WST-8 assay, respectively. A 24 h-treatment of HepG2 cells with 9-methylstreptimidone **(A)**, doxorubicin and daunorubicin hydrochloride **(B)**, 8α-hydroxy verrucarin A **(C)**, and chrysomycin A **(D)**, resulted in decreased H_2_S production (upper panels) without decreasing cell viability, except at the highest concentrations tested (lower panels). Comparison of each condition with DMSO, one-way ANOVA with Dunnett’s *post-hoc* test: **p < 0.01; ***p < 0.001, ****p < 0.0001.

Among the other compounds identified, eight of them are known to inhibit pro-survival pathways (Table 1 and Supplementary Table 4). Among these compounds, 9-methylstreptimidone, an inhibitor of NF-κB (Wang et al., 2006; Ishikawa et al., 2009) and possibly of the Akt/GSK3β pathway (Brassesco et al., 2012; Koide et al., 2015), showed a strong capacity to rescue yeast growth on a medium without methionine (Supplementary Table 4). Similarly, 8α-hydroxy verrucarin A and chrysomycin A, both inhibitors of NF-κB and of the Akt/GSK3β pathway (Deeb et al., 2016; Liu et al., 2016), also counteracted the growth defect phenotype induced by Cys4p overexpression (Supplementary Table 4). In order to check whether these drugs were also able to inhibit CBS activity in mammalian cells, we tested some of the most active compounds on H_2_S production in HepG2 cells. As shown in Figure 3, inhibitors of NF-κB and/or Akt/GSK3β pathway such as 9-methylstreptimidone (Figure 3A), 8α-hydroxy verrucarin A (Figure 3C) and chrysomycin A (which has been identified twice in the screening) (Figure 3D) were able to decrease H_2_S production (upper panel), although 8α-hydroxy verrucarin A and chrysomycin A also affected cell viability (lower panel). Similarly, two members of the anthracycline antibiotic family, doxorubicin and daunorubicin hydrochloride decreased H_2_S production in HepG2 cells (Figure 3B, upper panels) although they induced cell toxicity at higher concentrations (Figure 3B, lower panels). Finally, several small molecules with moderate activity were obtained in the screening, including several derivatives of naphtoquinones, which are also inhibitors of the NF-κB pathway (Supplementary Table 4). However, as their activity was moderate in rescuing cell growth in yeast, they were not tested on H_2_S production in HepG2 cells.

**FIGURE 4:**
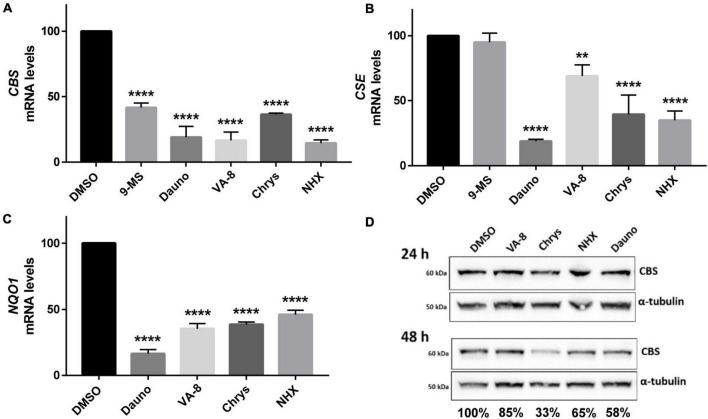
Effect of molecules identified in the pharmacological screening on *Cystathionine beta synthase (CBS)*, *cystathionine γ lyase (CSE)* and *NQO1* mRNA and *CBS* protein levels. **(A–C)** The level of *CBS* **(A)**, *CSE* **(B)**, and *NQO1* mRNAs: three genes involved in the defense against oxidative stress and which are regulated by the NF-κB and the Akt/GSK3β pathways, was assessed by RT-qPCR 24 h after treatment with 30 μM of 9-methylstreptimidone, 150 nM of daunorubicin hydrochloride, 10 nM of 8α-hydroxy-verrucarin A (VA-8), 200 nM of chrysomycin (Chrys) and 50 μM of NHX in HepG2 cells. **(D)** Similarly, the level of CBS protein was assessed by western-blot 24 and 48 h after treatment with the same drugs. Ratios of CBS/α-tubulin signals are indicated below each lane for 48 h fater treatment. **(A–C)** Comparison of each condition with DMSO, one-way ANOVA with Dunnett’s *post-hoc* test: **p < 0.01; ****p < 0.0001. Note that there is no error bar for DMSO because the different values we obtained were expressed as percentages compared to the DMSO condition which was set up at 100%.

To further confirm the hypothesis of a regulation of CBS activity by the Akt/GSK3β and/or NF-κB pathways, we assessed the impact of inhibitors of these pathway (9-methylstreptimidone, 8α-hydroxy verrucarin A, chrysomycin and daunorubicin) on the mRNA levels of *CBS*, *CSE* and *NAD(P)H quinone oxidoreductase 1* (*NQO1*), three genes known to be regulated by these pathways (Wang et al., 2014; Mutter et al., 2015; Ozaki et al., 2018). We also evaluated the effect of nitroxolin (NHX), a copper chelator that we previously identified as a candidate inhibitor for CBS (Conan et al., 2022) and which has been reported to reduce Akt phosphorylation and decrease GSK3β phosphorylation (Xu et al., 2019; Veschi et al., 2020). As shown in Figures 4A–C, all the compounds that reduced H_2_S production in HepG2 cells (Figure 3) also decreased mRNA levels of *CBS*, *CSE*, and *NQO1* genes, after 24 h of treatment. Accordingly, the levels of CBS protein were also reduced after 48 h but not after 24 h of treatment (Figure 4D). Altogether, these data suggest that the Akt/GSK3β and NF-κB pathways are involved both in the regulation of CBS activity (as assessed by the decreased H_2_S production 24 h after treatment), but also in the regulation of CBS expression, effect which is observed at the mRNA level 24 h after treatment and at the protein level 48 h after treatment.

## Discussion

### DYRK1A positively regulates CBS activity

**FIGURE 5:**
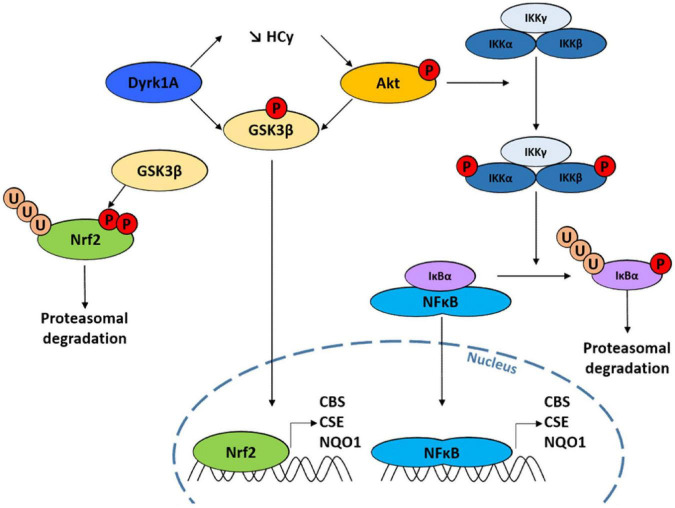
Scheme illustrating the genetic connection between Dual specificity tyrosine phosphorylation Regulated Kinase 1A (DYRK1A), GSK3β and Cystathionine beta synthase (CBS) through the Akt and NF-κB pathways.

Several studies have previously suggested a genetic interaction between *CBS* and *DYRK1A* but the nature of this interaction was still undetermined. Indeed, most of these data have been obtained in mouse models and it is sometimes difficult to assess in a complete organism whether the effects observed are direct or indirect consequences of *DYRK1A* or *CBS* deregulation. Here our data obtained in simple cellular models suggest that DYRK1A positively regulates CBS activity and that this relationship is conserved between yeast and human. This observation is consistent with previous findings obtained in mouse. For example, the triplication of both *DYRK1A* and *CBS* results in additive effects on hyperactivity and locomotion compared to mice having a triplication of either gene (Maréchal et al., 2019). Similarly, Delabar et al. (2014) have described increased CBS activity in the liver of *Dyrk1A*-overexpressing mice. This group also showed that forced expression of *Dyrk1A* (using an adenoviral construct) in the liver of *CBS+/−* mice induced increased CBS activity both in the liver (Tlili et al., 2013; Latour et al., 2015) and the brain (Baloula et al., 2018). The pathways involved in this regulation have been explored: decreased homocysteine levels, resulting from *Dyrk1A* overexpression, induced increased phosphorylation of the serine threonine kinase Akt (on Serine 473) (Guedj et al., 2012; Abekhoukh et al., 2013), which is then activated to promote cell survival by inhibiting apoptosis. On the contrary, mice or rats with hyperhomocysteinemia (resulting from a high-methionine diet) have decrease Dyrk1A protein levels both in the liver and the brain (Hamelet et al., 2009; Rabaneda et al., 2016) and consequently decreased phosphorylation of Akt (Liu et al., 2010, 2011; Figure 5). Our data, obtained in a simple model organism, thus confirm previous observations suggesting a link between DYRK1A, homocysteine levels and Akt phosphorylation in one hand and between DYRK1A expression and CBS activity on the other hand.

### GSK3β negatively regulates CBS activity

The transcription factor NF-E2-related factor 2 (Nrf2) encodes a protein which has a short half-time of only 30 min and which stability is controlled through the regulation of its turnover by the ubiquitin-proteasome system. It controls the expression of over 100 genes, including *CBS* (Hourihan et al., 2013; Liu et al., 2020), *CSE* (Jamaluddin et al., 2022), and *NQO1* (Mutter et al., 2015), three genes that play an important role in cell response to oxidative stress. The homolog system to Nrf2 in yeast is Yap1p, which also confers protection against oxidative stress and regulates the expression of yeast homologs of *CBS* and *CSE* (Orumets et al., 2012). The kinase GSK3β has been shown to prevent the transcription of Nrf2 targets by phosphorylating two serine residues in Nrf2, leading to its nuclear exclusion and degradation (Salazar et al., 2006; Rada et al., 2011). All these data thus point toward a mechanism of regulation of CBS expression at the transcriptional level involving the Nrf2 pathway. This is consistent with our results showing that (i) the expression of Mck1p, the yeast homolog of GSK3β, antagonizes the effect of *CYS4*-OE in yeast (Figures 2A–B) and (ii) the expression of a constitutively active form of GSK3β decreased H_2_S production in HepG2 cells (Figure 2G). Interestingly, both kinase DYRK1A and Akt have convergent effects by directly inactivating GSK3β by phosphorylation at Thr356 (for DYRK1A) (Song et al., 2015) and on serine 9 (for Akt) (Tlili et al., 2013; Latour et al., 2015; Figure 5).

### Role of NF-κB and Akt/GSK3β pathways in the regulation of the expression and activity of CBS

The compounds identified in our screening also confirm the importance not only of the Akt/GSK3β pathway but also of NF-κB, which is often activated by Akt/GSK3β (Ozes et al., 1999), in the regulation of CBS activity and expression. NF-κB is a key regulator of genes involved in the response to inflammation and stress. Ordinarily, NF-κB is sequestered in the cytoplasm through a direct interaction with a member of the IκB family of inhibitor proteins such as IκBα. Diverse range of stimuli, including oxidative stress, lead to the activation of the IKK complex (which contains two IκB kinases, IKKα and IKKβ). Phosphorylation of IκBα by the IKK complex triggers its recognition by an E3 ligase complex which leads to its polyubiquitination and subsequent degradation by the proteasome. The liberated NF-κB dimer then translocates to the nucleus, where it binds specific DNA sequences, inducing the transcription of genes such as *CBS* (Li et al., 2012; Zhang et al., 2013), CSE (Wang et al., 2014; Ozaki et al., 2018), and *NQO1* (Dinkova-Kostova and Talalay, 2010). Supporting these observations, we observed that several of the compounds identified in our screen and known to inhibit Akt and/or NF-κB pathways (9-methylstreptimidone, 8α-hydroxy verrucarin A, chrysomycin…) induced a significant decrease in *CBS, CSE*, and *NQO1* mRNA levels (Figure 4). Similar results were obtained for NHX (Figure 4), a metal chelator that we previously identified as a candidate inhibitor for CBS (Conan et al., 2022), and which has been reported to reduce both Akt and GSK3β phosphorylation (Xu et al., 2019; Veschi et al., 2020). However, it is important to note that we cannot completely rule out possible additional effects on CBS at the post-transcriptional level. Indeed, several post-translational modifications, such as phosphorylation, glutathionylation or sumoylation, have been reported to play a role in the regulation of CBS enzymatic activity. In addition, CBS protein levels have been reported to be increased following Akt activation, without affecting *CBS* mRNA expression (Zhu et al., 2022), which suggests that these pathways may also target CBS enzymatic activity in addition to its expression. This is also suggested by the fact that H_2_S production was decreased 24 h after treatment with 9-methylstreptimidone, 8α-hydroxy verrucarin A, chrysomycin and daunorubicin, whereas the level of CBS protein was not reduced before 48 h of treatment. This suggests that these pathways act at the level of both CBS enzymatic activity and expression level.

Concerning the identification of *MUP1*, *MMP1*, *STP2*, *UBP7*, and *UBP11* genes in the genetic screen, all five suggest a common role in overexpressing amino acid permeases at the plasma membrane, possibly to capture the low traces of methionine present in the medium and due to possible incomplete purification of other amino acids added in the medium. Indeed, Ubp7p and Ubp11p have been shown to deubiquinylate permeases ubiquitinylated by Rsp5p (the homolog of Nedd4L in yeast), preventing their endocytosis (Tardiff et al., 2013; Weinberg and Drubin, 2014). However, the fact that these genes also mitigate the cytosolic acidification of *CYS4*-OE cells suggest a possible other molecular mechanism. A possible mechanism to explain these results could be that an import of extracellular leucine or methionine would activate the Target Of Rapamycin (TOR) signaling pathway (Takahara et al., 2020; Vellai, 2021), which in turn inhibits the retrograde response, the pathway which regulates the response to oxidative stress and which is the equivalent of the NF-κB pathway in yeast (Johnson and Johnson, 2014).

### Role of metal chelation in the regulation of CBS activity

Several compounds identified in our drug screening point toward an important role of metal ion chelation in the regulation of CBS activity, as previously reported (Maréchal et al., 2019; Conan et al., 2022). Indeed, we previously showed that decreasing intracellular copper levels decreased the effects of *CBS* overexpression in yeast and H_2_S production in HepG2 cells (Conan et al., 2022). Here, the identification of several compounds involved in metal chelation [4-(2-Thiazolylazo)-resorcinol, 1-(2-Thiazolylazo)-2-naphtol, γ-thujaplicin, doxorubicin and daunorubicin hydrochloride, naphtoquinones…] confirm the importance of this process to decrease CBS activity. Several studies have shown that exposure of HepG2 cells or neuronal-like SH-SY5Y cells to copper (and to a lesser extent Zinc) induce phosphorylation of Akt and GSK3β (Ostrakhovitch et al., 2002; Walter et al., 2006; Barthel et al., 2007; Hickey et al., 2011) and that copper chelation reduces the levels of activated Akt and thus of inactivated GSK3β (Guo et al., 2021). Accordingly, we previously reported that several copper chelators such as D-penicillamine, trientine and several members of the 8-hydroxyquinoline family [clioquinol, chloroxine, NHX, and PBT2 (2-(dimethylamino) methyl-5,7-dichloro-8-hydroxyquinoline)] efficiently decreased CBS activity in several cell lines (Conan et al., 2022). Although further investigation is needed, the data obtained here with NHX (Figures 4A, D) suggest that the mechanism of action of this compound involves, at least in part, decreased CBS activity through the NF-κB and Akt/GSK3β pathways.

Disulfiram (DSF) is another compound that we previously identified in a similar drug screening (Maréchal et al., 2019). Its mechanism of action has not been fully deciphered. It has been suggested that DSF could inhibit CBS by its ionophore activity, increasing intracellular copper levels, as Cu-DSF was found more active than DSF on its own (Zuhra et al., 2020; Supplementary Figures 2A, B), which seemed in disagreement with our finding that increasing copper levels increased CBS activity (Conan et al., 2022). The relationship we here describe between the Akt and NF-κB pathways and CBS regulation could bring an explanation to this apparent discrepancy. Indeed, several groups have shown in various cellular models that DSF, on its own or combined to copper, decreases Akt phosphorylation (Kim J. Y. et al., 2016; Park et al., 2018; Nasrollahzadeh et al., 2021; Zha et al., 2021) as well as the NF-κB pathway (Wang et al., 2003; Guo et al., 2010; Yip et al., 2011). These data are in agreement with the observation that DSF on its own decreases H_2_S production, although with a limited effect, but has an increased effect (as well as increased toxicity) when combined to copper (Supplementary Figures 2A, B). Accordingly, we observed decreased *CBS* (as well as *CSE* and *NQO1*) mRNA levels in HepG2 cells treated with DSF-Cu (Supplementary Figure 2D). On the contrary, incubation with Bathocuproine disulphonate (BCS), a copper chelator totally abolished the effect of DSF (Supplementary Figure 2C).

### Extrapolation of these findings to the context of Down syndrome

Our results, supported by others in mouse models (Tlili et al., 2013; Delabar et al., 2014; Latour et al., 2015; Baloula et al., 2018; Maréchal et al., 2019), suggest that the overexpression of *DYRK1A* and *CBS* have additive effects and that CBS expression and/or activity is increased following DYRK1A overexpression. On the contrary, Dyrk1A inhibition results in decreased CBS expression and/or activity as shown by H_2_S production in HepG2 cells treated with EGCG. This suggests that therapeutic research focusing on DYRK1A inhibition should, at least in part, also take care of the problem of CBS triplication, at the condition that these inhibitors are specific of DYRK1A and do not inhibit GSK3β as well, as our data suggest that GSK3β activation is needed to reduce Nrf2 activity and thus CBS expression.

Several studies have shown that the expression and/or activity of CBS is tightly regulated and strongly depends on the redox state of the cell (Mosharov et al., 2000; Banerjee and Zou, 2005), meaning that the triplication of this gene would not necessarily mean an overexpression. However, CBS overexpression has been extensively reported in patients with DS (Kamoun et al., 2003; Ichinohe et al., 2005; Panagaki et al., 2019). Accordingly, increased oxidative stress, confirmed in several studies in these patients, as well as a hyperactivation of the PI3K/Akt/GSK3β pathway have been reported in the brain of DS patients (Perluigi et al., 2014). All these data suggest that any molecule decreasing the level of oxidative stress, inhibiting NF-κB pathway and/or activating GSK3β activity may result in decreasing CBS expression. Although several preclinical studies and clinical trials aimed at reducing oxidative stress using various anti-oxidant molecules have been performed (Rueda Revilla and Martínez-Cué, 2020), none of them has looked at the level of CBS expression and/or activity. This would be worth further investigating.

However, the regulation of CBS expression and/or activity also depends on other genes of chromosome 21. We showed here that *DYRK1A* overexpression increases CBS activity. Several other genes present on chromosome 21, including *SOD1* and *APP* are directly or indirectly involved in mitochondrial function, contributing to oxidative stress (Izzo et al., 2018) and may thus on their own or in combination with others have an impact on CBS expression and/or activity. On the contrary, *RCAN1*, also present on chromosome 21, encodes an inhibitor of the NF-κB pathway and its overexpression may then be expected to decrease CBS activity and/or expression.

Taken together, our data provide further insights into the regulation of CBS activity and into the relationship with other genes important for brain development and functioning such as *DYRK1A* and GSK3β. Although further studies are still needed to fully understand the different contributions of these molecular actors into the pathophysiology of DS, the hope is that they will lead to a better understanding of the molecular mechanisms underlying the pathology of DS, and thus to the development of more effective therapy that will bring amelioration or prevention of cognitive deficits in people with DS.

## Data availability statement

The original contributions presented in this study are included in the article/Supplementary material, further inquiries can be directed to the corresponding author.

## Author contributions

PC, AL, FB, CV, and GF: conceptualization. PC, AL, NC, FB, OM, CV, and GF: formal analysis. GF: funding acquisition and project administration. PC, AL, NC, CR, LC, and JM: investigation. PC, AL, FB, OM, CV, and GF: methodology. OM, CV, and GF: resources. OM, FB, CV, and GF: supervision. PC, AL, NC, CR, LC, CV, and GF: validation and visualization. PC, AL, and GF: writing—original draft preparation. PC, CV, and GF: writing—review and editing. All authors commented on the different versions of the manuscript, and read and approved the final manuscript.
