# Supplementary material for: New insights into the regulation of Cystathionine beta synthase (CBS), an enzyme involved in intellectual deficiency in Down syndrome
Source: Front Neurosci. 2023 Jan 9;16:1110163. doi: 10.3389/fnins.2022.1110163 (PMC9879293; doi:10.3389/fnins.2022.1110163)
Supplement: Supplementary file 1 [file Data_Sheet_1.docx]

**Supp Table 1**: *S. cerevisiae* yeast strains used in study

| **Strain** | **Genotype** | **Source** |
| --- | --- | --- |
| **S288C strains** |  |  |
| BY *WT* | BY4743 *MATa/α* *his3Δ1/his3Δ1 leu2Δ0/leu2Δ0 met15Δ0/MET15 LYS2/lys2Δ0 ura3Δ0/ura0* | Euroscarf |
| *yak1*Δ | BY4742 (*MATα* *ura3Δ0 leu2Δ0 his3Δ1 lys2Δ0*) *vma1::kanMX4* | Euroscarf |
| **W303 strains** |  |  |
| W303 *WT* | *MATa leu2-3,112 trp1-1 can1-100 ura3-1 ade2-1 his3-11,15* | Lab collection |
| *mck1*Δ | W303 except *mck1::kanMX4* | This study |

**Supp Table S2:** primers used in this study

| **Application** | **Primer sequences** |
| --- | --- |
| Sequencing clones of the genetic screening  *MCK1* deletion  in yeast  *CYS4*-FL subcloning  *YAK1* subcloning  in pRS416-*GPD*  *MCK1* subcloning  in pRS426-*TEF*  *MUP1* subcloning  in pRS416-*GPD*  *STP2* subcloning  in pRS416-*GPD*  *MMP1* subcloning  in pRS416-*GPD*  *UBP7* subcloning  in pRS426-*TEF*  *UBP11* subcloning  in pRS426-*TEF*  *YAK*1 K398R mutant  *MCK1* K68R mutant | 5’-GTGCTGCAAGGCGATTAAGT-3’  5’-TGTGGAATTGTGAGCGGATA-3’  5’-AATTTTCTTTTTATTTTCCGAAACCCCCACTCCATCACATTCTAGTACATCGGAT  CCCCGGGTTAATTAA-3’  5’-TTGTTCATTAAATTTTCCGAGGGGAAAGAGAACAAATTAATAGAAAATTAGAAT  TCGAGCTCGTTTAAAC-3’  5’-CGGGATCCCGATGACTAAATCTGAGCAGCAAG-3’  5’-GCCTCGAGTCTTATGCTAAGTAGCTCAGTAAATCC  5’-TCCCCCGGGGGAATGAACTCATCCAATAATAACG-3’  5’-CCCTCGAGGGTTATTCTTCGACAATGTGAAG-3’  5’-TCCCCCGGGGGAATATGTCTACGGAAGAGCAG-3’  5’-CCCTCGAGGGTTATTCAGCAACTTTCGTAGG-3’  5’-TCCCCCGGGGGAATGTCGGAAGGAAGAACG-3’  5’-CCCTCGAGGGGGTTACAGCGATTTTTCTTG-3’  5’-TCCCCCGGGGGACATGCCTATCTTATCACTATC-3’  5’-CCCTCGAGGGCCTTAAAATTCTATCCCATAAG-3’  5’-TCCCCCGGGGGAATGGATGAATTTGAATCTACC-3’  5’-CCCTCGAGGGAGTACTTCACTAAGTTAACAG-3’  5’-TCCCCCGGGGGAAATGCTAGACGATGATAAGG-3’  5’-CCATCGATGGTTGCTAGTCATAAACCCTTTC-3’  5’-TCCCCCGGGGGACAATGTTATTAAACCCAGATC-3’  5’-CCCTCGAGGGGATGCAATTAACAGAATTCTTC-3’  5’-TGCTGACGAAAGAGATATTGGCTGTAAGAGTGGTTAAATCGA-3’  5’-TCGATTTAACCACTCTTACAGCCAATATCTCTTTCGTCAGCA-3’  5’-GGGCCCCTTTGCAATTAGAAAAGTCCCTGCTCAT-3’  5’-ATGAGCAGGGACTTTTCTAATTGCAAAGGGGCCC-3’ |

**Supp Table S3:** primers used for qPCR

| **Gene** | **Primer sequences** |
| --- | --- |
| CBS  CSE  NQO1  GAPDH | 5’-GTCATCTACAAGCAGTTCAAACAGA-3’  5’-GCTGTGGTACTGGATCTGCTC-3’  5’-GGCCTGGTGTCTGTTAATTGT-3’  5’-GCCATTCCGTTTTTGAAATGCT-3’  5’-CAGCGGCTTTGAAGAAGA-3’  5’-GCAGGGTCCTTCAGTTTA-3’  5’-GAGTCAACGGATTTGGTCGT-3’  5’-TTGATTTTGGAGGGATCTCG-3’ |

**Supp Table S4:** Drugs from NIH chemical libraries that were able to rescue the growth of *CYS4*-OE yeast cells on a methionine-free medium

| **Compounds** | **CAS number** | **Structure** | **Growth rescue**  **on Met-free medium** |
| --- | --- | --- | --- |
| **9-Methylstreptimidone**  NSC 248958 | 51867-94-8 | 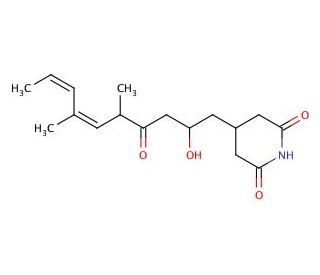 | 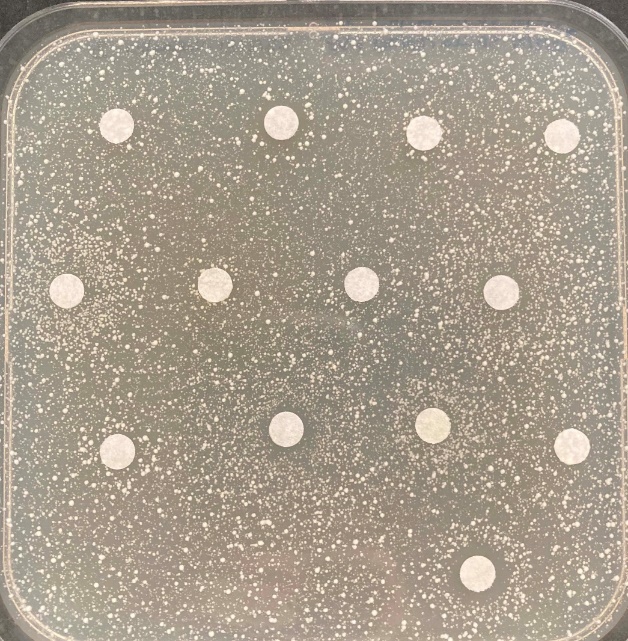 |
| **4-(2-Thiazolylazo)-resorcinol**  NSC 298197 | 2246-46-0 | 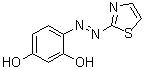 | 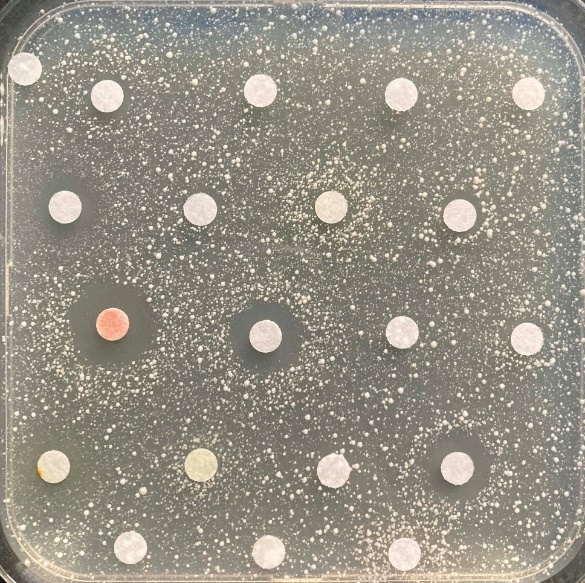 |
| **1-(2-Thiazolylazo)-2-naphtol**  NSC 139021 | 1147-56-4 | 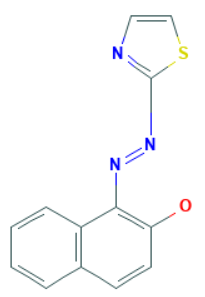 | 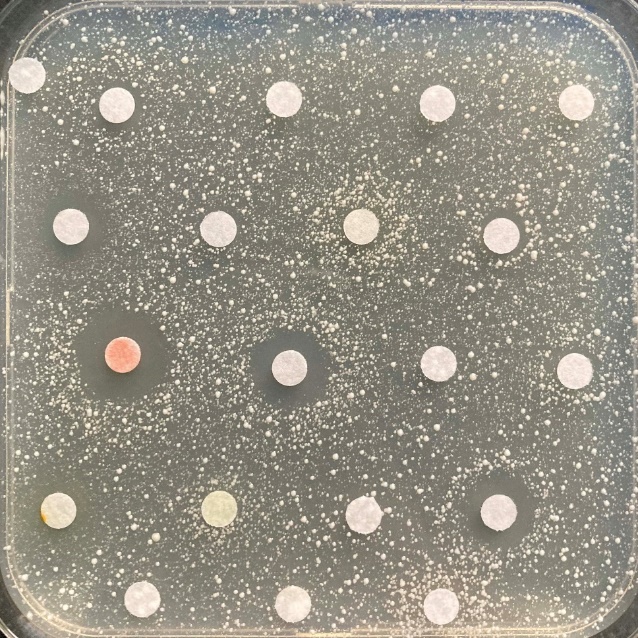 |
| **Zinc pyrithione**  NSC 68093 |  | 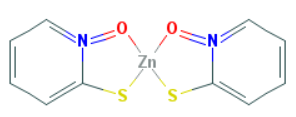 | 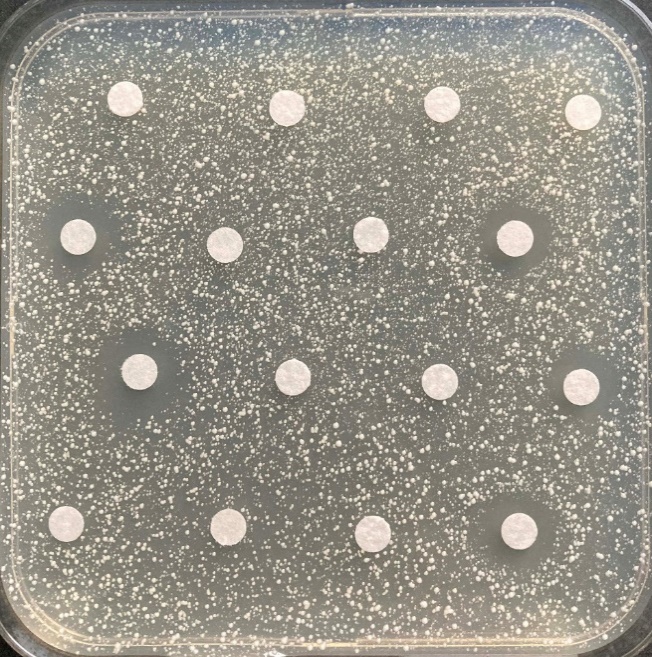 |
| **N,N-dimethyldaunomycin hydrochloride**  NSC 258812 | 70095-84-0 | 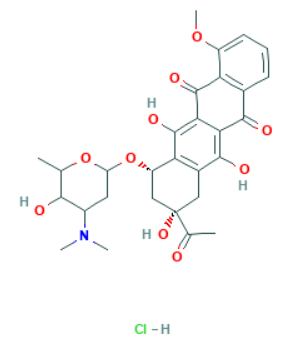 | 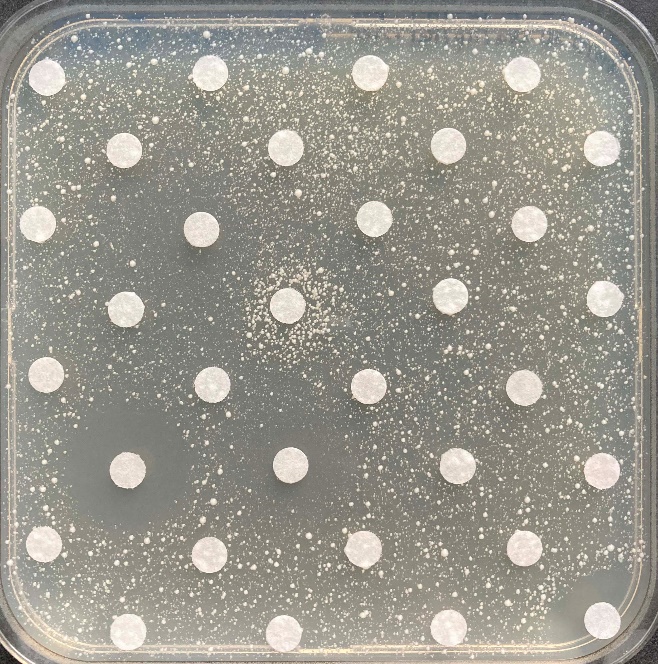 |
| **Doxorubicin dihydrochloride**  NSC 123127 | 25316-40-9 | 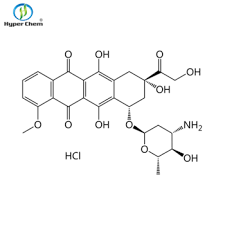 | 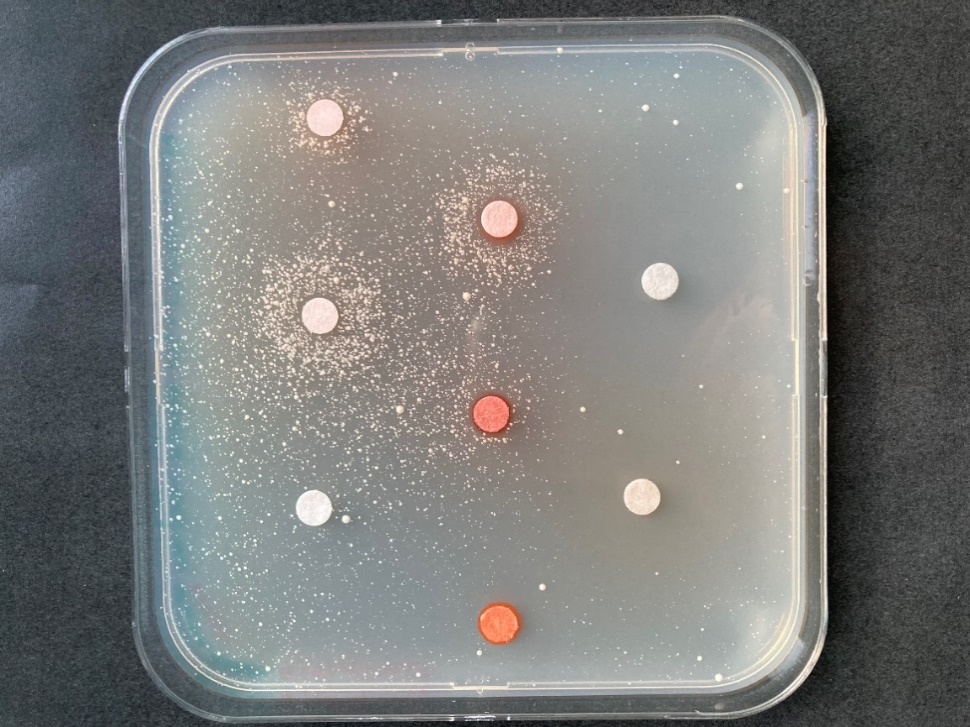 |
| **Daunorubicin hydrochloride**  NSC 82151 | 23541-50-6 | 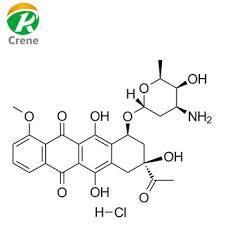 | 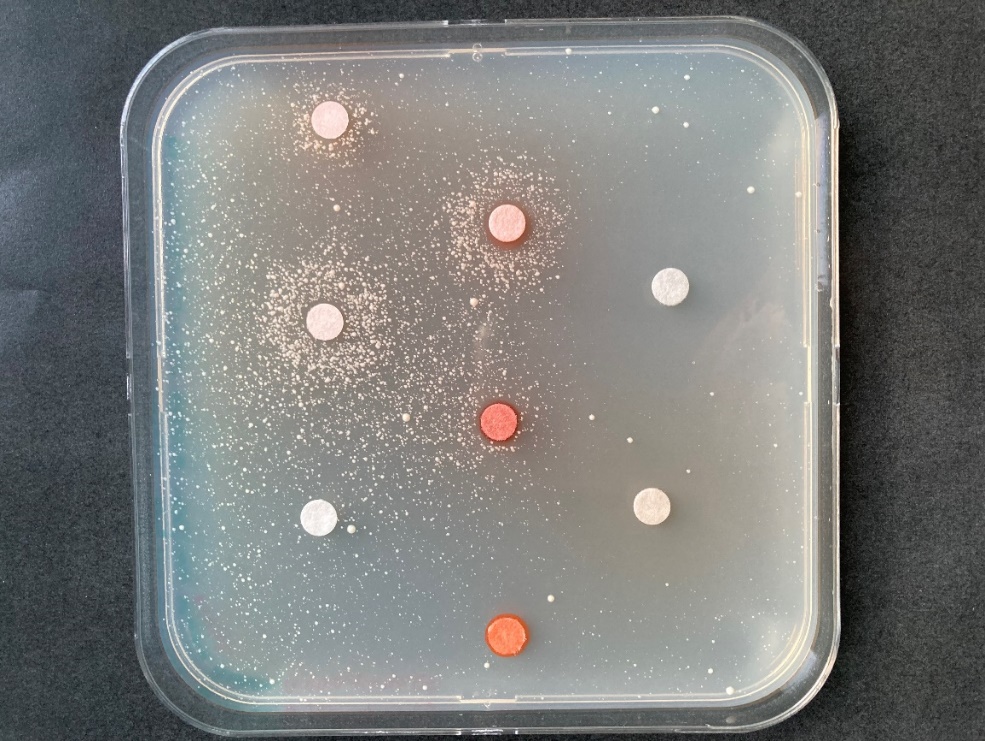 |
| **γ-thujaplicin**  (2-hydroxy-5- isopropyl-2,4,6-cyclo-heptatrienone)  NSC 18805 | 672-76-4 | 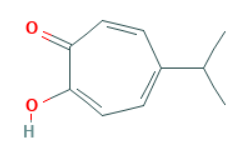 | 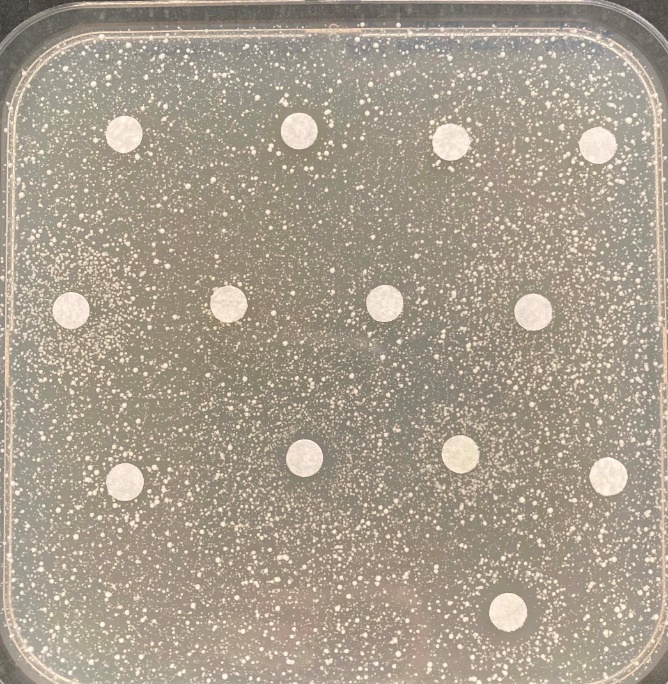 |
| **Verrucarin A,10-epoxide**  NSC 283445 | 74560-38-6 | 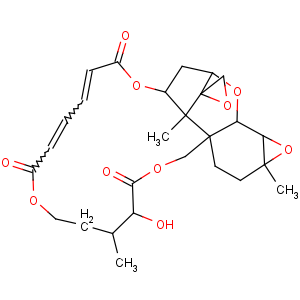 | 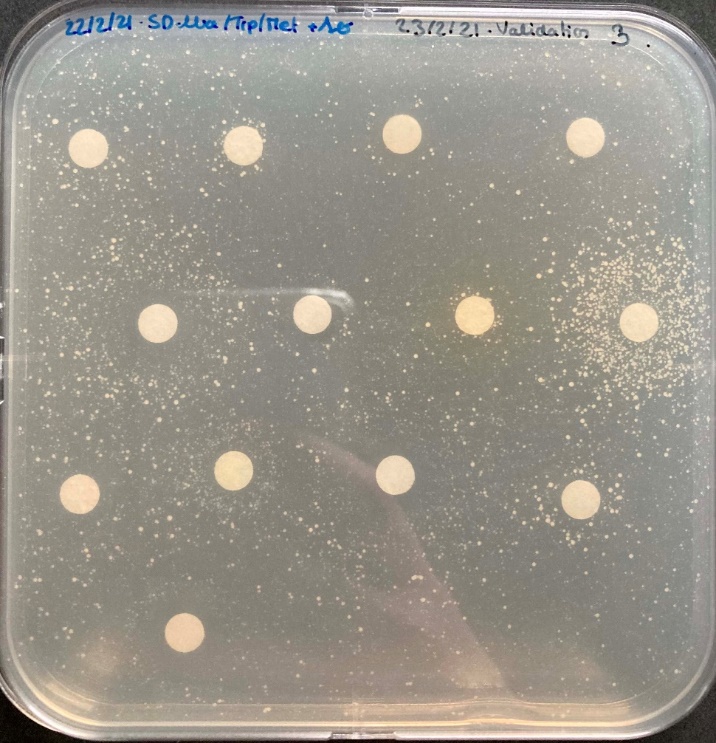 |
| **8α-Hydroxy-verrucarin A**  NSC 391312 | 74516-62-4 | 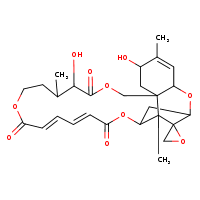 | 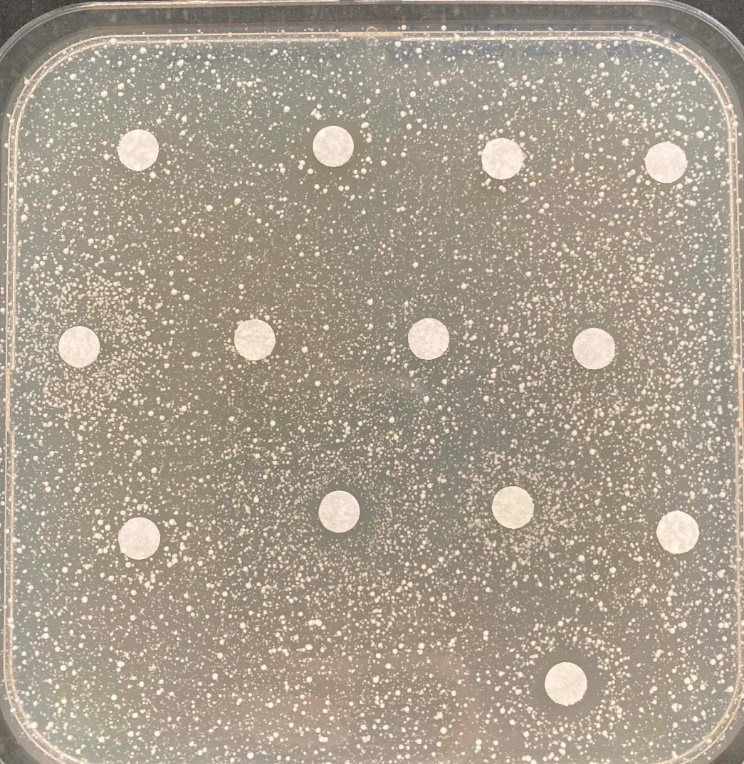 |
| **Monoacetyl verrucarin A epoxide**  NSC 292463 | 2841-82-9 | 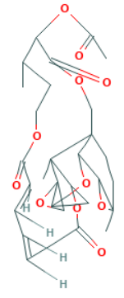 | 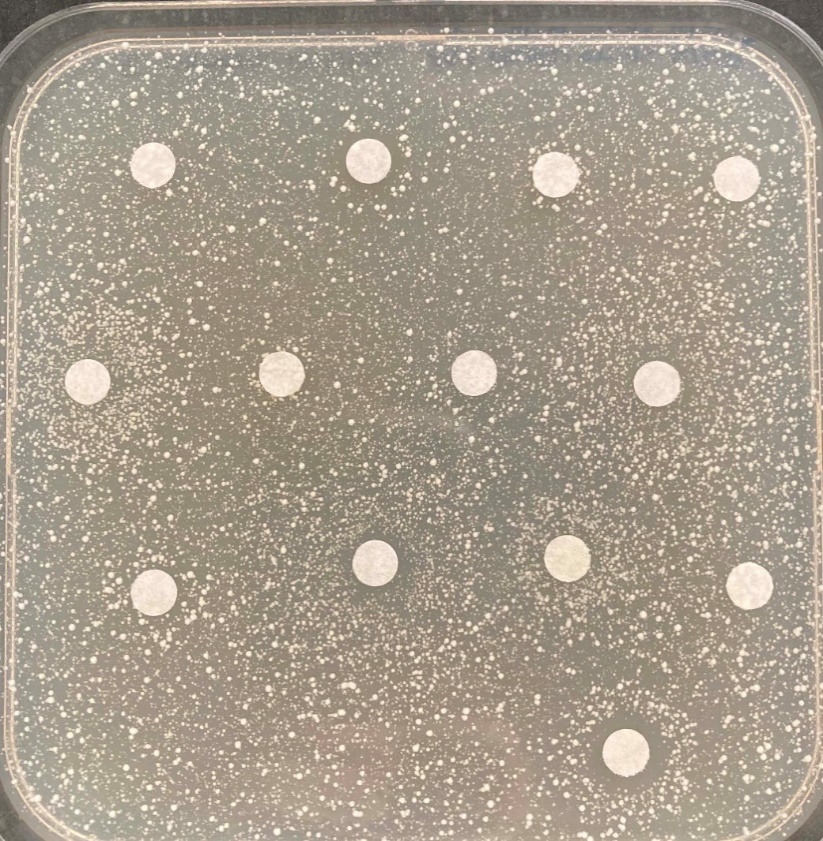 |
| **Chrysomycin A**  NSC 354844 | 82196-88-1 | 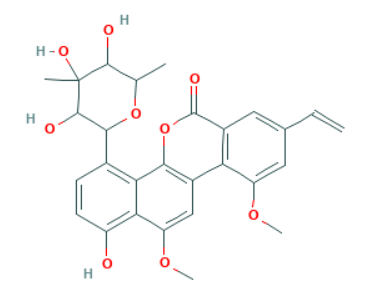 | 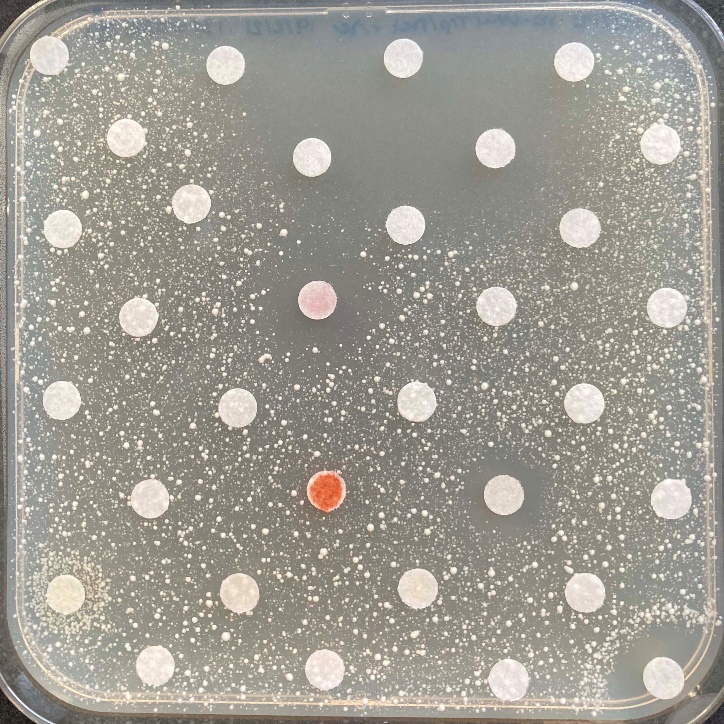 |
| **Chrysomycin A**  NSC 354844 | 92841-46-8 | 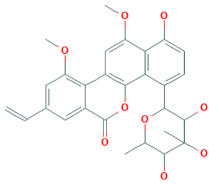 | 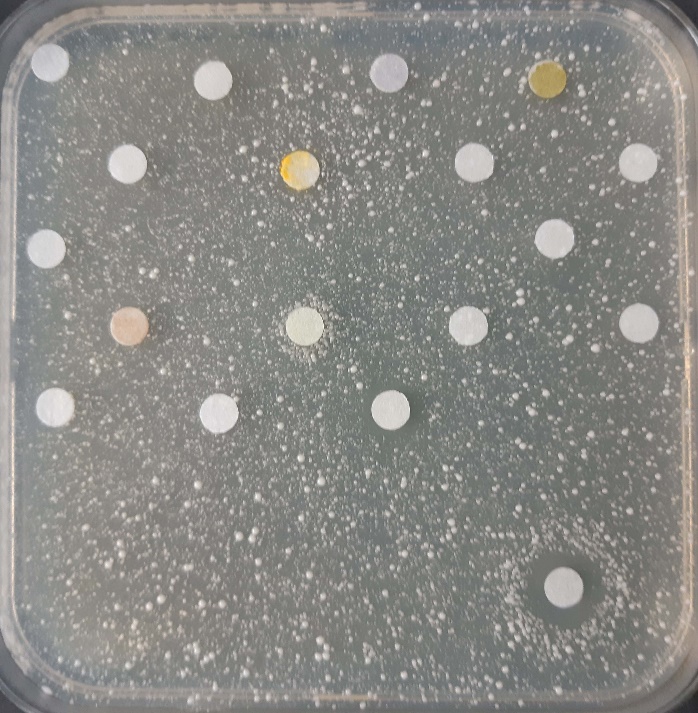 |
| **2-(5,11-dimethyl-6H-pyrido[4,3-b]carbazol-6-yl)ethyl benzoate**  NSC 163443 |  | 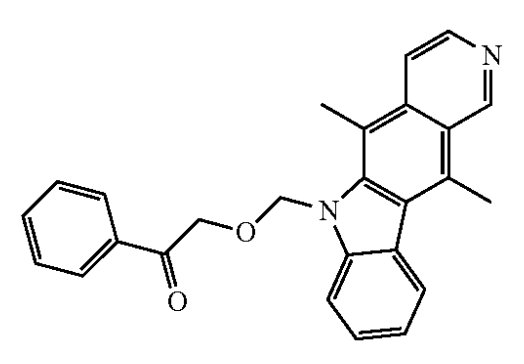 | 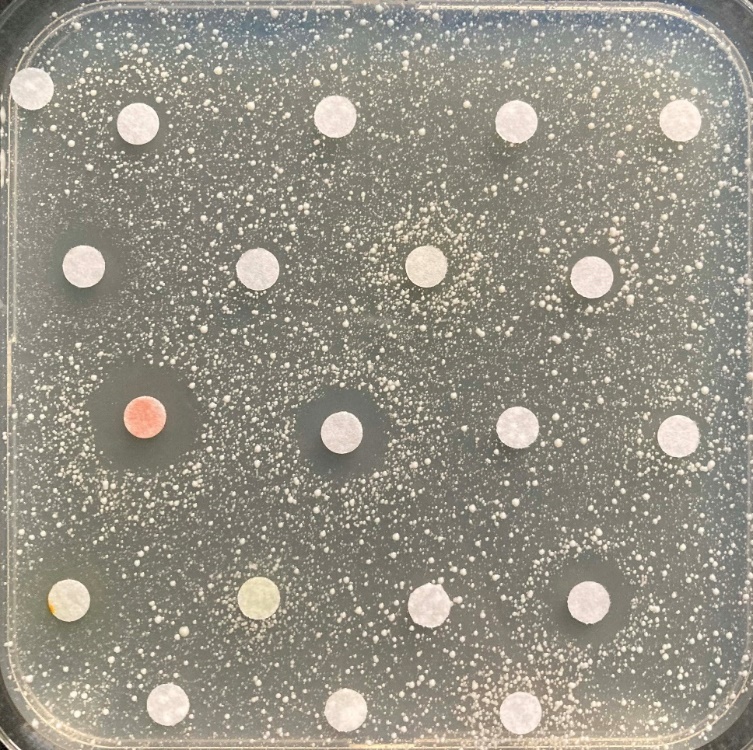 |
| **N,n-dimethyl-1,3-dioxo-isoindole-2-sulfonamide**  NSC 13800 | 5430-46-6 | 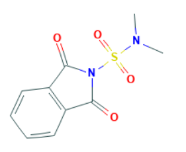 | 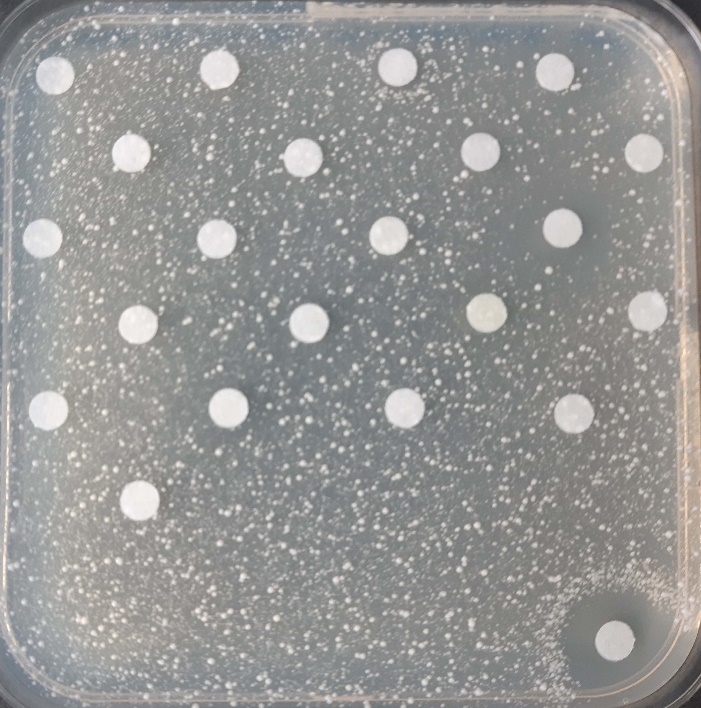 |
| **1,1,3-Tribromo-3-methyl-2-butanone**  NSC 1027 | 1578-05-8 | 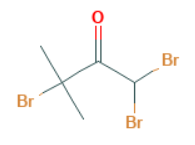 | 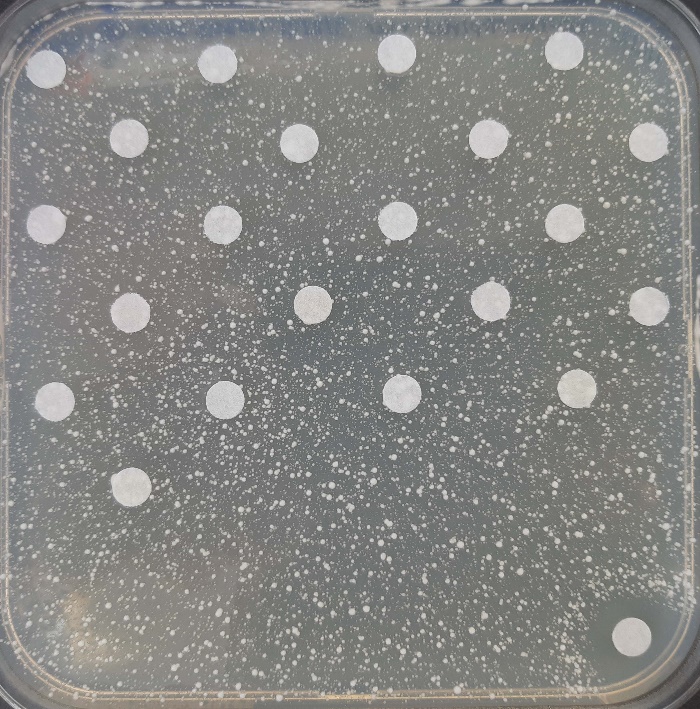 |
| **(2E)-3-Methyl-2-(methyl-imino)-2,3-dihydro-naphtho[2,3-d][1,3]thiazole-4,9-dione**  NSC 659501 |  | 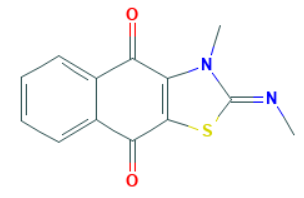 | 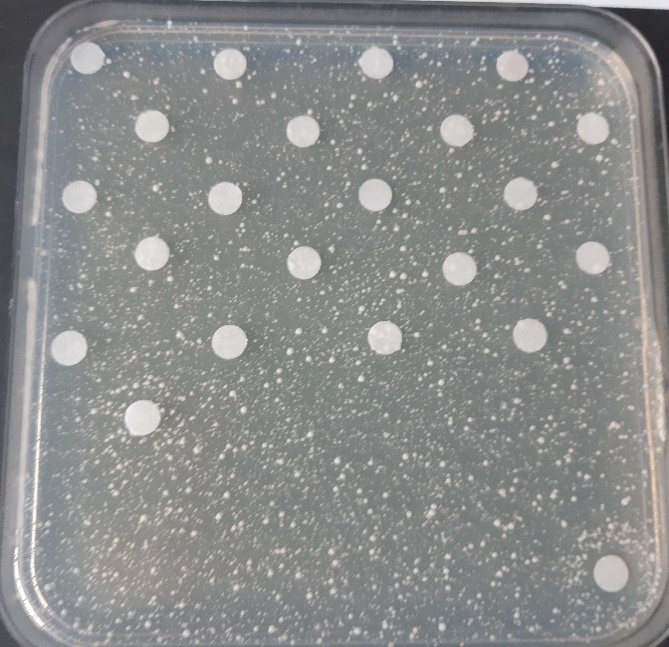 |
| **2,3-Dibromo-1,4-naphtho-quinone**  NSC 618332 | 13243-65-7 | 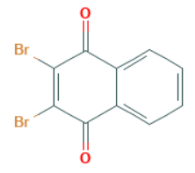 | 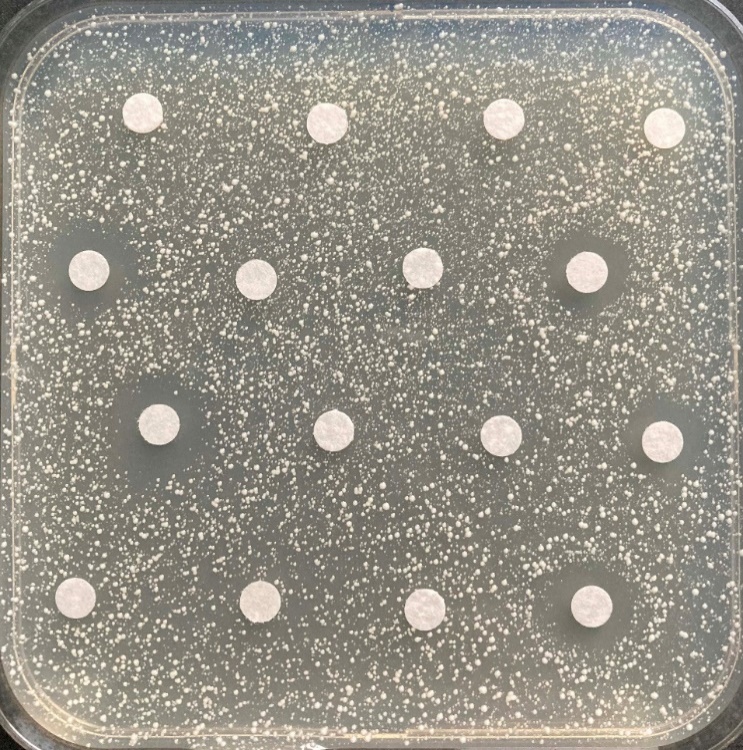 |
| **NSC 659997** |  | 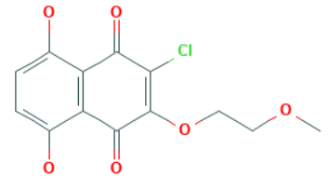 | 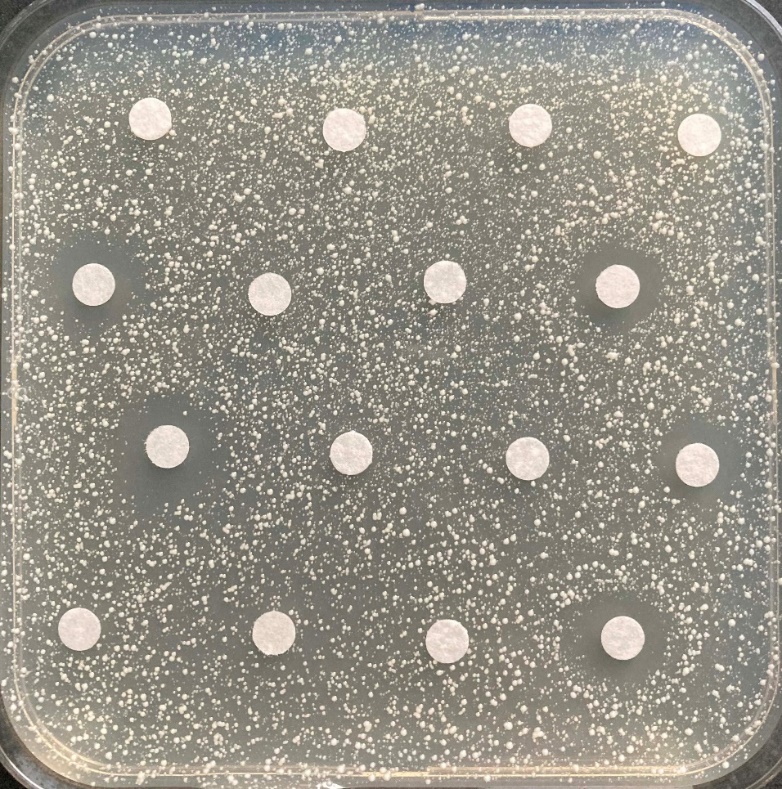 |
| **2-(3,4-dichloro-anilino)benzo[f][1,3]benzothiazole-4,9-dione**  NSC 631521 |  | 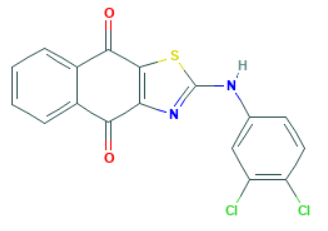 | 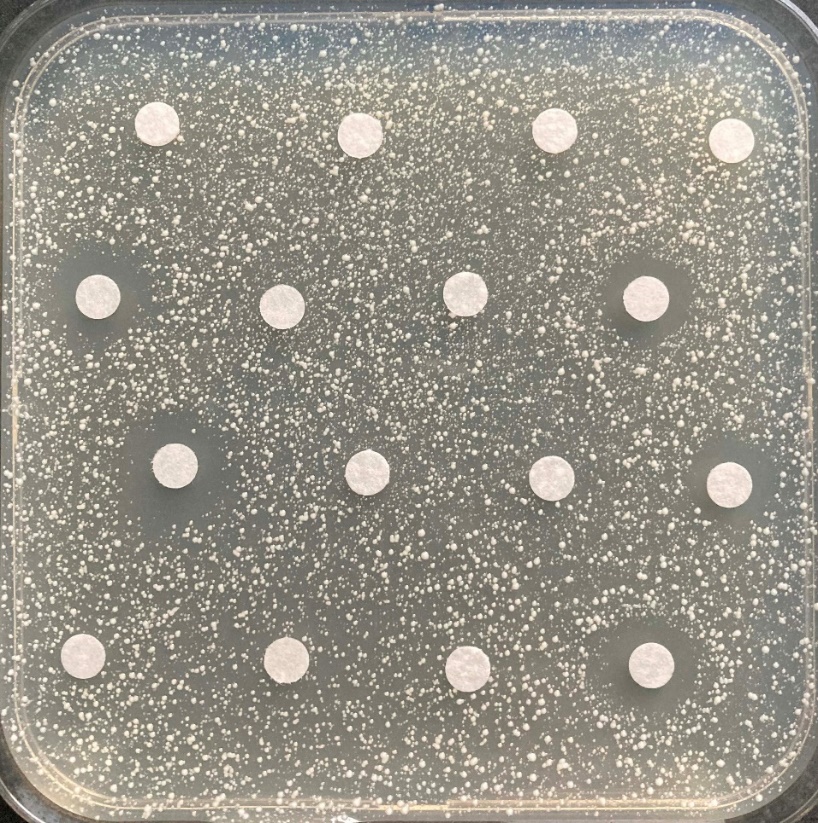 |
| **α-lapachone**  NSC 26327 | 4707-33-9 | 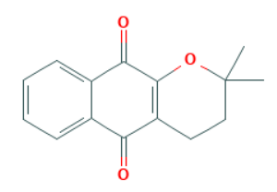 | 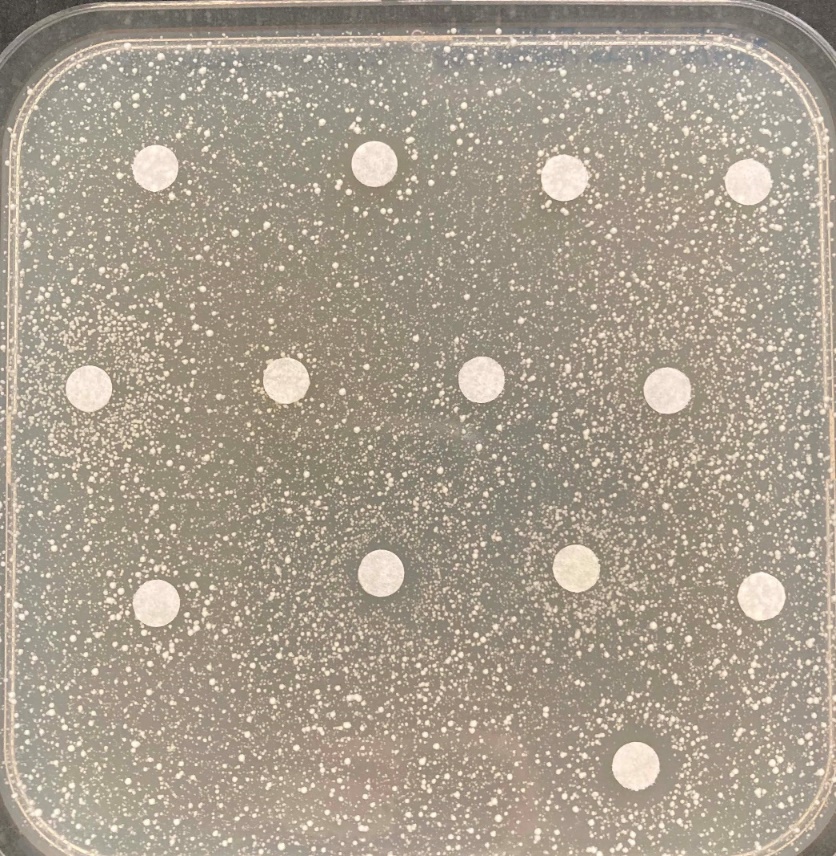 |

**Supplementary Figure 1**: **Validation of the genetic interactions between *CBS*, *DYRK1A* and *GSK3β* in yeast and human HepG2 cells**. **(A-B)** Effect of the genes identified in the genetic screening on cytosolic acidification induced by *CYS4*-OE. **(A)** Both *MMP1* or *MUP1* overexpression decrease the effects of *CYS*4-OE on cytosolic acidification. **(B)** Similarly, the overexpression of *UBP*7 or *UBP11* also mitigates the consequences *CYS4*-OE. **(C)** Wild-type or mutant Dyrk1A overexpression did not have any effect on cell viability 48 h after transfection, as assessed with the WST-8 assay. **(D)** A 24 hour-treatment of HepG2 cells with EGCG impacted cell viability at concentrations higher than 125 µM. **(E)** The expression of a constitutively active form of GSK3β (p.S9A) did not have any effect on cell viability 72 h after transfection. **(A, B, C, F)** Comparison of each condition with *CYS4*-OE for A and B, with empty vector for C and with DMSO for D, one-way ANOVA with Dunnett's post-hoc test: *****, p<0.0001.* **(E)** Student’s t test: ****, p<0.001.*

**Supplementary Figure 2**: **Effect of disulfiram (DSF) and the role of copper in its ability to decrease H_2_S production in HepG2 cells**. **(A)** A 24 hour-treatment with DSF alone decreases H_2_S production (upper panel) in a dose-dependent manner but also impacted viability, as assessed with the WST-8 assay (lower panel). **(B)** Addition of copper increased the ability of 10 µM of DSF to decrease H_2_S production (upper panel) but also strongly impacted cell viability as assessed with the WST-8 assay (lower panel). **(C)** Pre-incubation with 100 µM of Bathocuproine disulphonate (BCS), a copper chelator, abolished the effect of 12.5 µM of DSF on H_2_S production, confirming the importance of copper in DSF action. **(D)** Effect of 5 µM DSF + 1 µM CuCl2 on *CBS*, *CSE* and *NQO1* mRNA levels in HepG2 cells. Comparison of each condition with DMSO, one-way ANOVA with Dunnett's post-hoc test: **, p<0.05; **, p<0.01; ***, p<0.001, ****, p<0.0001.*
