# Supplementary figures and images for: New insights into the regulation of Cystathionine beta synthase (CBS), an enzyme involved in intellectual deficiency in Down syndrome
Source: Front Neurosci. 2023 Jan 9;16:1110163. doi: 10.3389/fnins.2022.1110163 (PMC9879293; doi:10.3389/fnins.2022.1110163)

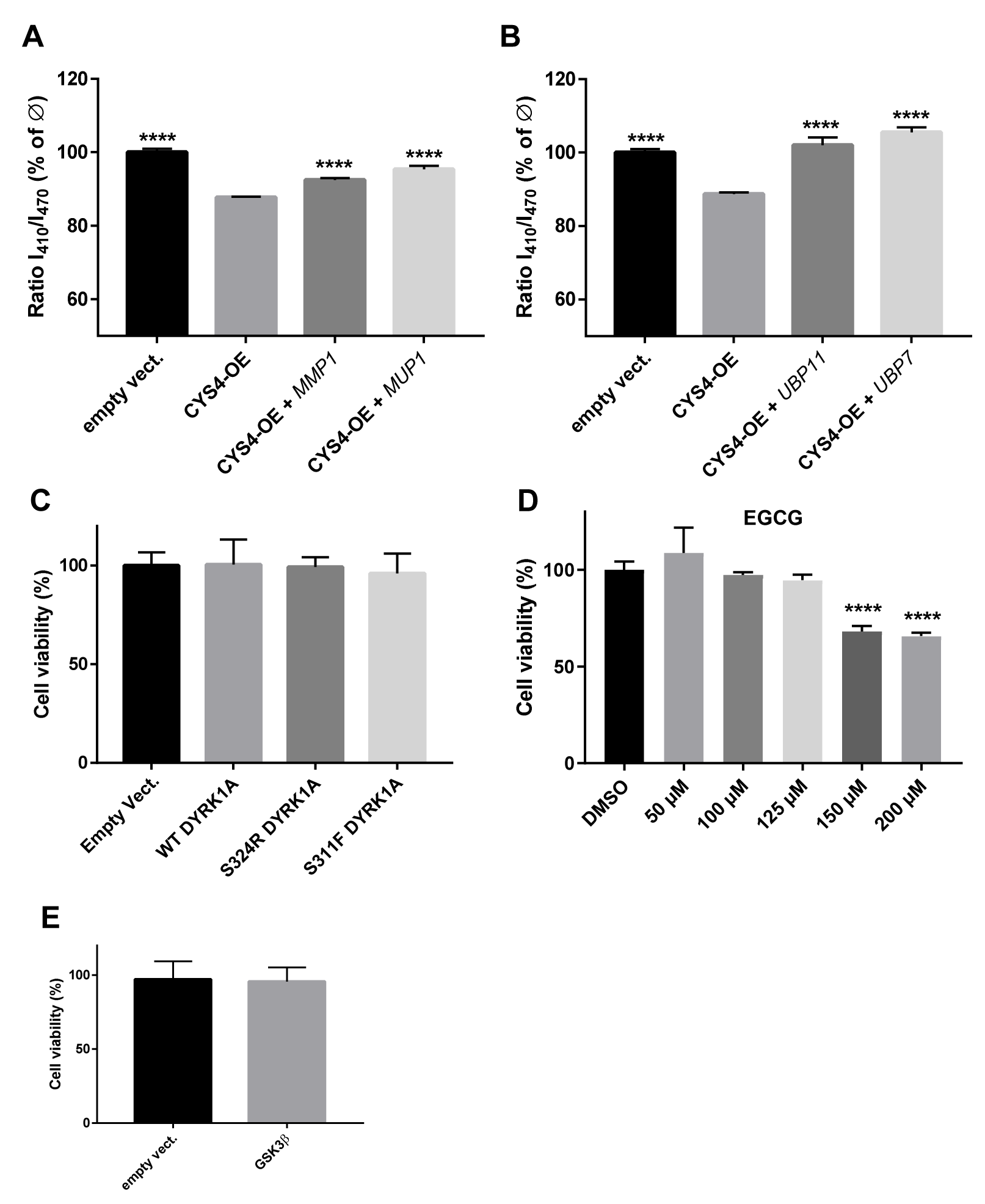

Supplement: Supplementary file 2 [file Image_1.TIF]

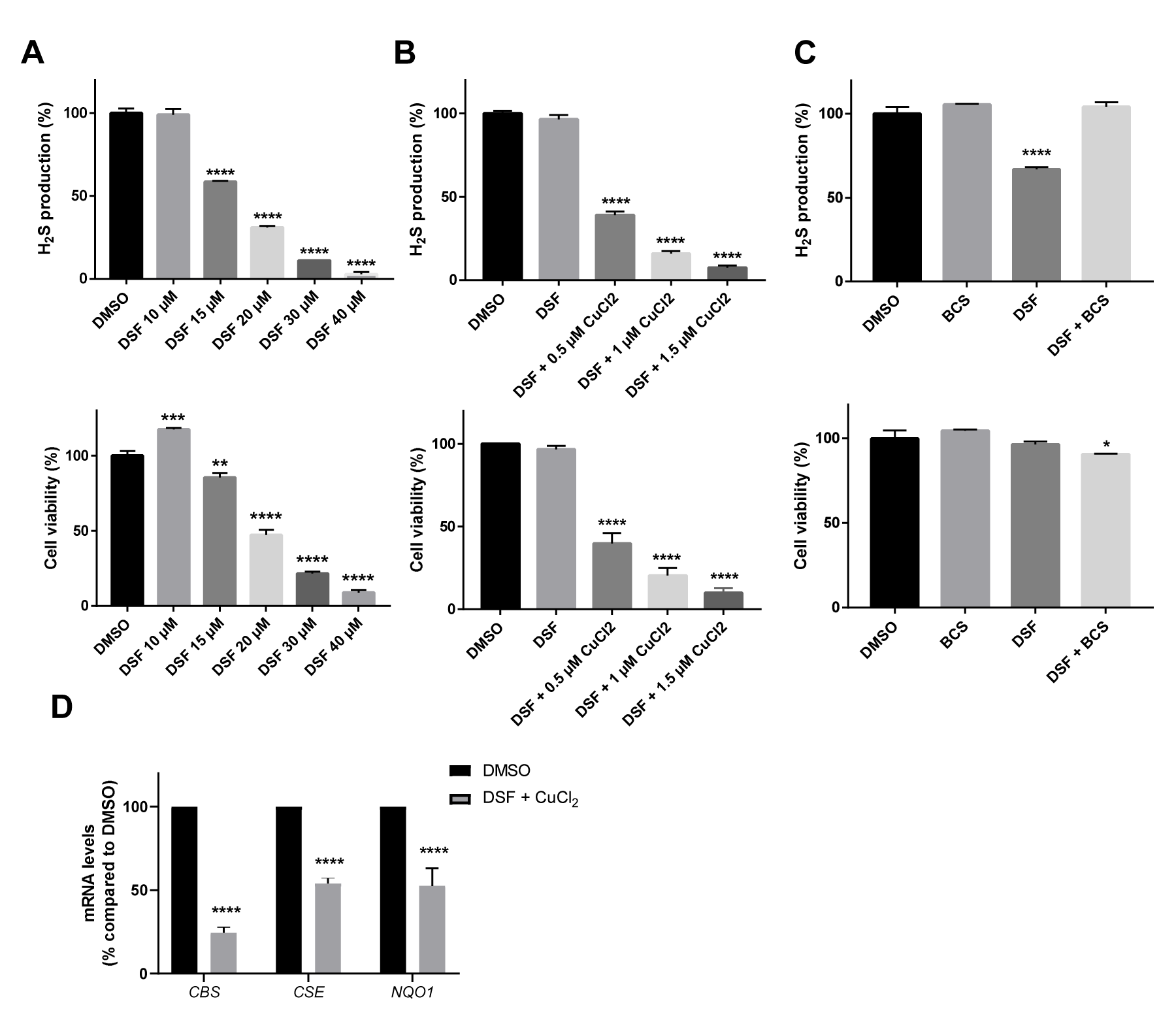

Supplement: Supplementary file 3 [file Image_2.TIF]
